# How Single Cells Form Shells: Maturation and Secretion of Lorica‐Forming Material in the Tintinnid *Schmidingerella* (Alveolata, Ciliophora)

**DOI:** 10.1111/jeu.70025

**Published:** 2025-07-08

**Authors:** Maximilian H. Ganser, Birgit Weißenbacher, Sabine Agatha

**Affiliations:** ^1^ Department of Environment and Biodiversity University of Salzburg Salzburg Austria

**Keywords:** biomaterial, electron energy loss spectroscopy, light microscopy, live observations, marine planktonic protist, transmission electron microscopy, ultrastructure

## Abstract

Tintinnid ciliates are distinguished by their loricae (shells), the key synapomorphy of these mainly marine planktonic protists. They can divide daily, with a considerable portion of biomass stored in the loricae. During each division, lorica‐forming material (LFM) is produced and afterwards used by the proter (anterior division product) to construct a new lorica, while the opisthe (posterior division product) retains the parental one. Many aspects of lorica formation remain unclear, and no study describes the entire process from material maturation via secretion to assembly. Here, we present the first thorough investigation at cellular and sub‐cellular levels, employing light microscopy on dividers and postdividers, as well as transmission electron microscopy on primarily cryofixed specimens from a *Schmidingerella* culture. Our study reconstructs LFM maturation, identifying two main developmental stages: the morula‐shaped precursor granules and the mature granules. The latter cluster in the proter's ventral portion with a peripheral longitudinal strip of small granules embedded in large ones. Ultrastructurally and chemically, the mature granules of both size classes are identical. Through detailed live observations, we followed and documented, for the first time, the process of cell division, the behavior of the proter, the release of LFM granules, and features of lorica formation.

## 1 Introduction

The formation of shells, loricae, or tests by protists is a fascinating process that links cellular behavior and biology with the self‐assembly mechanisms of biomaterials.

Tintinnids are unique among marine planktonic ciliates for their ability to form loricae. After each transverse fission, the opisthe (posterior division product) remains attached to the bottom of the lorica, while the proter (anterior division product) leaves and forms its own lorica. Lorica construction comprises the lorica‐forming material (LFM) production during the cell cycle, including its maturation and accumulation, followed by its secretion and ciliate‐mediated self‐assembly.

The process of maturation has not been characterized in any tintinnid species, except for fragmentary ultrastructural observations showing different types of intracellular granules assigned to the LFM (Hedin 1975; Laval 1972; Laval‐Peuto 1975; Laval‐Peuto et al. 1979; Sokolova and Gerassimova 1984; Wasik and Mikołajczyk 1992).

The site of LFM secretion in the proter remains uncertain despite several hypotheses (Biernacka 1965; Campbell 1927; Entz Jr. 1909; Kofoid 1930; Laval‐Peuto 1981). Upon contact with seawater, the secreted LFM becomes foamy, expands, and hardens (Campbell 1927). In a recent light microscopical study on the tintinnid *Schmidingerella*, the lorica wall finally exceeds the intracellular LFM quantity by at least 4.5‐fold (Agatha et al. 2025).

Formation of the lorica wall is thought to involve a ciliature‐mediated self‐assembly (Campbell 1927; Laval‐Peuto 1981). Previous observations on lorica formation are limited but suggest that the posterior lorica portion is produced first, followed by the anterior portion in a second phase (e.g., Laval‐Peuto 1981). Documentation and live observations of lorica formation are incomplete or entirely lacking.

Distinct lorica wall textures and structures across several tintinnid taxa have been revealed by electron microscopy (Agatha and Bartel 2022). Members of the genus *Schmidingerella* typically form transparent (hyaline) champagne flute‐shaped loricae with an alveolar wall and a posterior process (a narrowed posterior lorica elongation). Occasionally, however, they are capable of producing *Coxliella*‐shaped loricae, which are cylindroidal with a broadly rounded posterior end and a spiraled wall structure. A study applying histological staining techniques and enzymatic digestion experiments suggested a proteinaceous material as the main lorica component in tintinnid ciliates (Agatha and Simon 2012).

The present study is a continuation of Agatha et al. (2025) which focused on the cell division pattern as well as the timing of LFM production, the material accumulation, and the final amount of LFM available for lorica construction in *Schmidingerella*. Using the same monoclonal *Schmidingerella* strain from the Northeast Pacific, we here report on the process of intracellular LFM maturation, its subsequent secretion, and the formation of the lorica. To this end, we investigated the ultrastructure of cryofixed specimens, which offer an unprecedented preservation of membranes and cytoplasmic structures, and of chemically fixed specimens to ensure comparability with previously published transmission electron microscopy (TEM) data. Additionally, detailed live observations of the cell behavior during division, secretion, and lorica formation complemented our analyses.

## 2 Methods

### 2.1 Culture Material

Monoclonal culture material of the model tintinnid *Schmidingerella* collected from the Northeast Pacific was provided by Kelley Bright and Suzanne L. Strom from the Shannon Point Marine Centre, Western Washington University, USA, in November 2022. The strain investigated in the present study (SPMC 176) was maintained at our laboratory for several months in artificial seawater plus f/2 medium (Guillard 1975) at a salinity of 32‰, a pH of 7.8, a temperature of 15°C, and a light–dark cycle of 12:12 h. Neither conjugating specimens nor resting cysts were observed in the investigated strain. The dinoflagellate *Heterocapsa triquetra* (CCMP 448) and the haptophyte *Isochrysis galbana* were provided by the Strom Lab as a food source and maintained in culture as well.

We refrain from using a species name since the taxonomy of the genus *Schmidingerella* is unclear based on the available morphological and molecular data. Several *Schmidingerella* species with similar lorica morphologies apparently co‐occur in the Northeast Pacific (Smith et al. 2022). Furthermore, the few genetic data on *Schmidingerella* are rarely accompanied by detailed morphometric data, while morphologic studies (of the loricae) usually lack complementary molecular data. The *Schmidingerella* strain analyzed here has been sequenced; yet, the molecular data are the focus of a forthcoming study that is currently in progress.

### 2.2 Live Investigations

The observations of freely swimming specimens were conducted at room temperature (about 22°C) in 6‐well plates (CELLSTAR 6 Well Cell Culture Multiwell Plates, Greiner Bio‐one). Each well had a diameter of 3.5 cm and was filled with about 9.6 mL of water corresponding to a water depth of about 1 cm. We used an Olympus stereo microscope SZX16 equipped with an SDF Plan Apo 1 × PF objective at up to 115× magnification and dark field illumination. To minimize disturbances during cell division and the subsequent lorica formation process, an external LED light source (2700 K) was used instead of the internal dark field illumination. High‐resolution observations of specimens in vivo were conducted at up to 2000× magnification with an Olympus BX53 compound microscope, using bright field and differential interference contrast optics. More than 630 images and 134 videos were captured with an Olympus OMD E‐1 Mark II digital camera, using the OM capture software (OM Digital Solutions).

### 2.3 Transmission Electron Microscopy (TEM)

Previous volumetric analyses of the identical *Schmidingerella* strain (SPMC 176) revealed that lorica‐forming material (LFM) is first visible in middle divider stages and production continues until cell division with an accelerated rate in late dividers (Agatha et al. 2025). Accordingly, the entire LFM production process should be trackable in middle to late divider stages.

For chemical fixation, we selected middle and late dividers from strain SPMC 176 and subjected them to a solution of 5% glutaraldehyde in 0.05 M cacodylate buffer (1000 mOsmol, pH 7.8) with 2% osmium tetroxide for 1 h. Next, the fixed cells were washed three times in 0.2 M cacodylate buffer and stored in 70% ethanol at 4°C overnight. The subsequent dehydration was done with a graded ethanol series (concentrations of 70%, 80%, 90%, 96%, and three steps with 100% ethanol for 15 min each) followed by three steps in propylene oxide for 10 min each. The cells were embedded using increasing concentrations of agar low viscosity resin (ALVR; Agar Scientific, Stansted, UK), that is, ratios of propylene oxide to ALVR of 3:1 (for 1 h), 1:1 (2 h), and finally 1:3. Subsequently, the resin containing the cells was filled in small aluminum dishes and placed in a desiccator at room temperature for 4 d and finally in an oven at 40°C for 3 h and 60°C for 24 h for polymerization.

For cryofixation, late dividers from strain SPMC 176 were transferred with a minute amount of culture medium into sample holders with a depth of 200 μm and a diameter of 1.2 mm. Cryofixation was conducted by means of a Leica EM PACT High Pressure Freezer (Leica Microsystems, Vienna, Austria). Freeze substitution was performed with acetone containing 2% osmium tetroxide and 0.05% uranyl acetate, following Lütz‐Meindl and Aichinger (2004) and using a Leica EM AFS (Leica Microsystems, Vienna, Austria). After three or four washing steps with anhydrous acetone at room temperature (about 22°C), the samples were washed three times for 10 min each in propylene oxide before embedding in Agar Low Viscosity Resin (ALVR; Agar scientific, Stansted, UK). Embedding was performed as described above. Subsequently, the polymerization of the samples was achieved after 4 d in a desiccator at room temperature and 24 h in an oven at about 60°C.

Four cryofixed late dividers and eight chemically fixed middle and late dividers were sectioned by means of an ultramicrotome (Ultracut S; Reichert AG, Vienna, Austria) fitted with a diamond knife. Ultrathin sections (70 nm thick) were mounted on Formvar‐coated copper grids. The investigations were conducted with a Zeiss EM 910 transmission electron microscope (Karl Zeiss AG, Oberkochen, Germany). More than 3000 micrographs were taken with a Sharp:Eye digital camera system (Tröndle), using the computer software ImageSP Viewer (SysProg and TRS, Moorenweis, Germany).

### 2.4 Electron Energy Loss Spectroscopy (EELS)

TEM‐coupled EELS was performed to determine the relative content of carbon, oxygen, and nitrogen in LFM granules, using 40 nm‐ultrathin sections of two late dividers (one chemically fixed, one cryofixed) mounted on uncoated hexagonal narrow mesh copper grids. The analyses were carried out, using a Zeiss (LEO) 912AB Omega transmission electron microscope (Karl Zeiss AG, Oberkochen, Germany) with an in‐column energy filter and a LaB6 cathode operating at an acceleration voltage of 120 kV at 50,000× magnification. The measured area for EELS was defined by using a 100‐μm spectrometer entrance aperture.

First, wide range EELS measurements (up to 1073 eV) were conducted for an overview of the general elemental composition of the selected granules, using illumination angles of 2.5 mrad, exposure times of 30 s, and a 125× spectrum magnification. Second, measurements were performed near the specific energy loss edges of nitrogen, oxygen, and carbon, using illumination angles of 1.0 mrad, exposure times of 1–2 s, and a 125× spectrum magnification. For each EELS measurement, five integration cycles were acquired. The elements were identified via the N K‐edge at an electron energy loss of 401 eV, the O K‐edge at an electron energy loss of 532 eV, and the C K‐edge at an electron energy loss of 284 eV. The EEL‐spectra were captured, using a slow‐scan dual‐speed CCD camera TRS Sharp:Eye (Tröndle, Moorenweis, Germany) and the iTEM Software (Olympus SIS, Münster, Germany).

The measurements were performed at different sites: (1) three granule sites (in the centres of two small mature granules as well as in the center and the electron‐dense cap of one large mature granule) and the lorica wall of a cryofixed specimen; and (2) in two granule sites (one subunit of a morula‐shaped granule and a large mature granule) and the lorica wall of a chemically fixed specimen; the embedding medium was always used for reference.

### 2.5 Measurements

TEM micrographs of ultrathin sections from cryofixed late dividers and chemically fixed middle and late dividers, which showed the LFM cluster of mature granules in the proter as well as various morula‐shaped granules, were selected and processed in Adobe Photoshop (vers. 25.5.1). The granules were manually marked and the resulting images imported into Fiji (vers. 2.15.1; https://imagej.net/software/fiji/; Schindelin et al. 2012). Next, size measurements (Area, Centroid, Fit ellipse) of the marked granules were obtained with the “Analyze Particles” option resulting in two datasets, one for cryofixed specimens (“Cryo”) and a second one for chemically fixed specimens (“Chem”). Statistical analyses of the measurements were conducted in R (vers. 4.1.2), using RStudio (vers. 2023.12.1 + 402). For each dataset (Cryo and Chem), k‐means clustering was performed with the optimal number of clusters as determined by the gap statistic method (settings: K.max = 10, B = 500) implemented in the cluster package (vers. 2.1.2, https://doi.org/10.32614/CRAN.package.cluster). To ascertain whether granule measurements differed significantly within and between the two datasets, we performed an analysis of variance (ANOVA) followed by a Tukey's Honest Significance Difference (HSD) test for pairwise comparisons of group means for significant (*p* < 0.05) results. Subsequently, plots were generated with ggplot2 (vers. 3.5.0).

Series of three longitudinal thin sections each of one cryofixed and one chemically fixed late divider were analyzed to determine the distribution of the LFM granules in relation to the cell membrane: section 1 through the central portion of the LFM cluster and sections 2 and 3 with increasing distances to the central section.

The LFM cluster dimensions were measured in methyl blue‐eosin‐stained specimens (staining method detailed in Agatha et al. 2025) with the abovementioned light microscope at 800× magnification.

### 2.6 Terminology

The terminology of the oral and somatic ciliatures follows Agatha and Strüder‐Kypke (2007) and that of the division stages is according to Agatha et al. (2025). The term “morula” (“stech‐apfelförmige Gebilde”) was introduced by Entz Jr. (1935) and represents membrane‐bound electron‐dense compounds of polygonal structures. The term “mature granules” means cytoplasmic LFM granules ready for secretion.

## 3 Results

### 3.1 Genesis and Maturation of LFM

**FIGURE 1:**
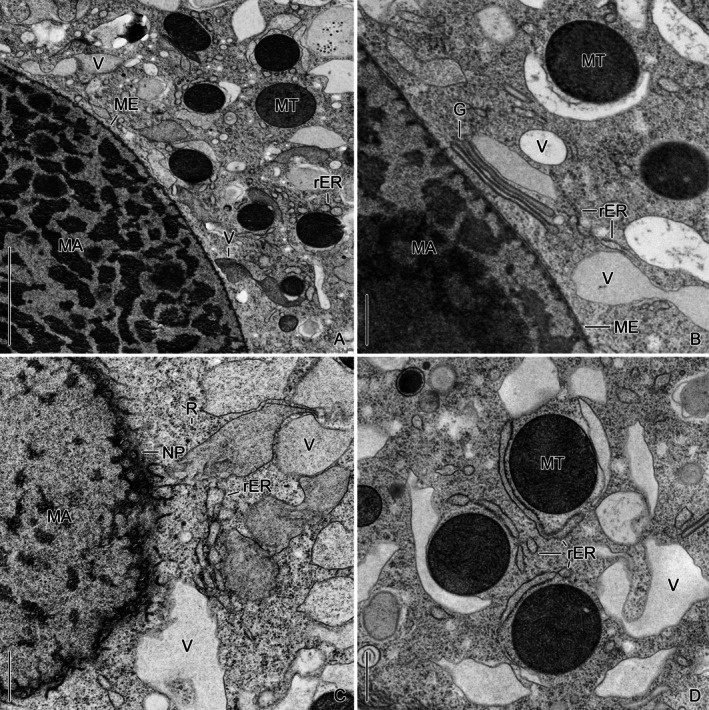
Ultrathin sections of cell organelles in cryofixed *Schmidingerella* dividers. The cells are sparsely equipped with Golgi bodies and a rough endoplasmic reticulum, which is mostly restricted to the vicinity of the macronuclear nodules and a bifacially differentiated type adjacent to the mitochondria. Free ribosomes are highly abundant and scattered in the cytoplasm. G, Golgi body; MA, macronuclear nodules; ME, macronuclear envelope; MT, mitochondria; NP, nuclear pores; R, free ribosomes; rER, rough endoplasmic reticulum; V, vesicles with fluffy or granular content. Scale bars 2 μm (A), 500 nm (B–D).

We first evaluated the endoplasmic reticulum and Golgi apparatus as sites for LFM genesis and found no evidence that this is the case. The rough endoplasmic reticulum is surprisingly sparse in the investigated early late and very late dividers, primarily consisting of single stacks with a luminal spacing averaging 92–119 nm (*n* = 14). These specimens were in phases of high lorica‐forming material (LFM) production or had already entered a phase of decreasing LFM production (Agatha et al. 2025). Membrane‐bound ribosomes are found on the rough endoplasmic reticulum close to the macronucleus, on bifacially differentiated endoplasmic reticulum adjacent to mitochondria (Figure 1D), and on the macronuclear envelope (Figures 1A,B and S4A). Furthermore, innumerable free ribosomes appear distributed in the cytoplasm (Figure 1C). Likewise, the Golgi apparatus comprises a comparatively low number of small Golgi bodies with only a few cisternal layers (Figure 1B).

Irregular vesicles are scattered in the cell and also close to the macronuclear envelope (Figures 1A–D, 3A,B,D–F and S1A, S4A). They measure on average 373–587 nm in width and 891–964 nm in length (*n* = 17 and 14; three images of same specimen analyzed). In dividers, the vesicles contain different materials: some enclose a fluffy content, while others contain loosely granular substances. In chemically fixed cells, such vesicles are hardly detectable because the membranes are less well preserved (Figure S1B).

**FIGURE 2:**
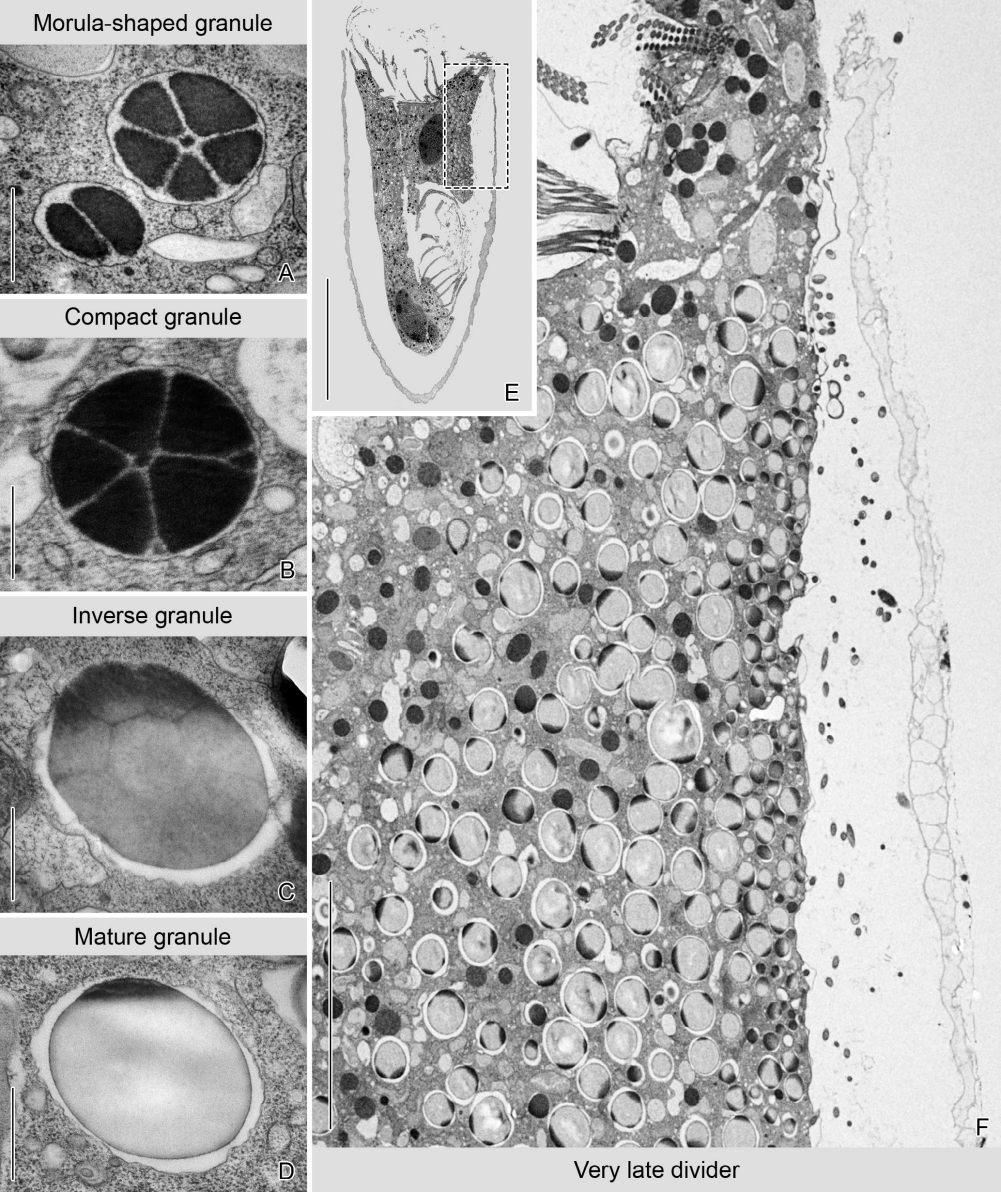
Ultrathin sections of cryofixed *Schmidingerella* late dividers. (A–D) LFM granule stages. (E, F) Cluster of mature granules in the proter. Dashed rectangle (E) denotes position of longitudinal section (F). Scale bars 500 nm (A–D), 50 μm (E), 10 μm (F).

The LFM is first recognizable as distinct granules scattered in the whole cell of early middle dividers, indicating that their production is not restricted to a certain cell region (see also Agatha et al. 2025). We describe the four maturation stages of LFM granules (morula‐shaped, compact, inverse, and mature granules) based on ultrastructural data in the following section (Figure 2).

**FIGURE 3:**
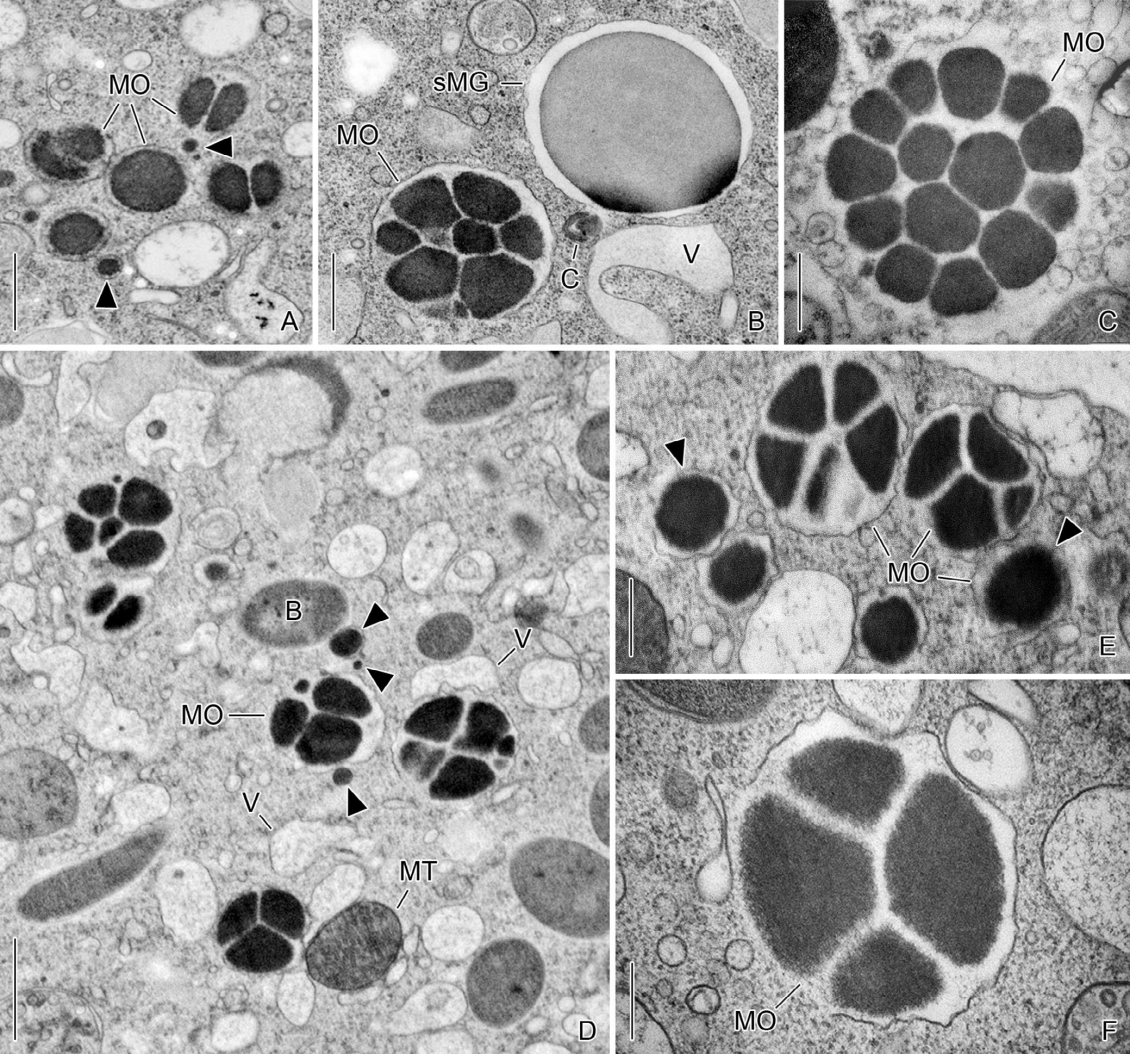
Ultrathin sections of mainly morula‐shaped lorica‐forming material granules in cryofixed (A, B, D–F) and chemically fixed (C) *Schmidingerella* late dividers. The size of the granules increases with the number of their constituting subunits, and the chemically fixed granules are on average significantly larger and have more subunits than the cryofixed ones (Figures S2, S3 and Tables S1, S2). Arrowheads mark electron‐dense aggregates. B, bacterium; C, capsule (tintinnid extrusome); MO, morula‐shaped granules; MT, mitochondrium; sMG, small mature granule; V, vesicles. Scale bars 500 nm (A–C, E), 250 nm (F), 1 μm (D).

Morula‐shaped stages were morphometrically analyzed in cryofixed specimens (three early late and one very late divider; Figures 2A, 3A,B,D–F and S1A, S8A; Table S1) and in chemically fixed specimens (one late middle and one early late divider; Figures 3C and S1B, Table S1). Frequently, morula‐shaped granules occur in small clusters (Figures 3A,D,E and S1A,B, S8A), particularly near the macronuclear nodules and the oral primordium (Figure S1B). They are often found in proximity to the vesicles and the endoplasmic reticulum (Figures 2A and 3D). Occasionally, electron‐dense aggregations are present adjacent to the morula‐shaped granules (Figure 3A,D,E). However, evidence of vesicle budding, fusion, or material sorting was not found.

In cryofixed specimens, the morula‐shaped granules are membrane‐bound, broadly ellipsoidal, and 883 ± 295 × 698 ± 239 nm in size (Table S1). Usually, 4.5 ± 2.4 subunits are recognizable. The individual electron‐dense subunits are separated from each other and from the frequently undulated surrounding membrane by an electron‐transparent space (Figures 2A, 3A,B,D–F). Tendentially, the maximum dimension of the morula‐shaped granules increases with the number of composing subunits (Figure S2), that is, those with a single sectioned subunit are 156–587 nm (410 ± 131 nm; *n* = 8) across, whereas the largest morula‐shaped granule measures 1749 nm and comprises 10 polygonal subunits. In chemically fixed morula‐shaped granules, the subunits have the same electron density as in cryofixed cells (Figures 3C and S1B); yet, the granules measure 1382 ± 518 × 1263 ± 471 nm and show 12.3 ± 5.1 polygonal electron‐dense subunits in a thin section (Table S1). Again, their size increases with the number of subunits (Figure S2). The chemically fixed granules are significantly larger (by about 17%; Figure S3) and have significantly more subunits (by 162%) than those of the cryofixed material (Figure S2). Furthermore, they are mostly globular. The surrounding membrane is destroyed and thus fragmentary (Figure 3C).

In the compact morula‐shaped granules, the distances between the electron‐dense subunits are much smaller than in the previous stage (Figures 2B and S4A). In contrast to the cryofixed compact granules, those of the chemically fixed dividers are not separated by an electron‐transparent space from the surrounding membrane and consist of more subunits (Figures S1B and S4B). Very rarely, chemically fixed granules combine features of the compact morula‐shaped granules (reticulate pattern of thin electron‐transparent lines) with characters of mature granules, namely, a protrusion and electron‐transparent spots (Figure S4C).

Inverse morula‐shaped granules were only detected in cryofixed dividers (Figure 2C) and are seemingly rare. They have electron‐dense caps with an uneven surface and are still separated by an electron‐transparent space from the surrounding membrane. Their abutting subunits are more electron‐transparent in their centers than at their surfaces, generating a reticulate pattern of electron‐dense lines.

**FIGURE 4:**
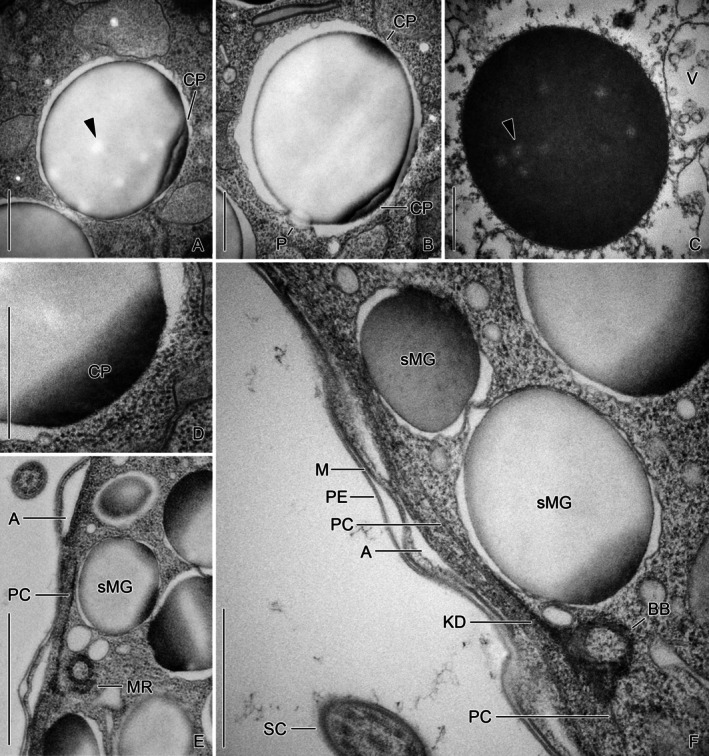
Ultrathin longitudinal sections of mature granules in cryofixed (A, B, D–F) and chemically fixed (C) *Schmidingerella* late dividers. (A, B) The cryofixed mature lorica‐forming material granules are amorphous and electron‐transparent. They have bright inclusions (arrowhead) and an electron‐dense surface and caps. Frequently, protrusions are visible. (C) The chemically fixed granules are amorphous and electron‐dense, except for some bright inclusions (arrowhead). (D) The cap of a mature granule in detail. In this region, the enclosing membrane is often invisible. (E, F) The mature granules cluster just underneath the lateral ciliary field whose monokinetids have the tintinnid‐specific associated structures (Agatha et al. 2022; Gruber et al. 2018). A, alveoli; BB, basal body; CP, electron‐dense cap; KD, kinetodesmal fiber; M, cell membrane; MR, extraordinary microtubular ribbon I; P, protrusion; PC, postciliary ribbons; PE, perilemma; SC, somatic cilium; sMG, small mature granules; V, vesicle. Scale bars 500 nm (A–D, F), 1 μm (E).

The mature granules of cryofixed specimens vary in size, but not ultrastructurally (Figures 2D,F, 3B, 4A,B,D–F, 6A and S1A). The electron‐dense reticulate pattern is no longer visible, resulting in a rather amorphous content which is, compared to the previous stage, considerably more electron‐transparent and contains some even more electron‐transparent regions (Figures 4A and S8B). Only the granules' surface and caps (one to four in a single section) remain electron‐dense. The surrounding membrane is closely associated with the granule in the cap regions (Figure 4D), while it appears undulated and separated by an electron‐transparent space in the remaining vesicle portions. The granules are frequently adjacent to vesicles containing fluffy or loosely granular material as well as to relatively indistinct Golgi bodies.

In chemically fixed late dividers, the mature granules also vary in size and are amorphous (Figures 4C and S7A). However, they have a much higher electron density, except for somewhat more electron‐transparent inclusions. Mature granules in cryofixed and chemically fixed dividers show small and electron‐transparent protrusions (usually one in a section; Figures 4B and S1B).

**FIGURE 5:**
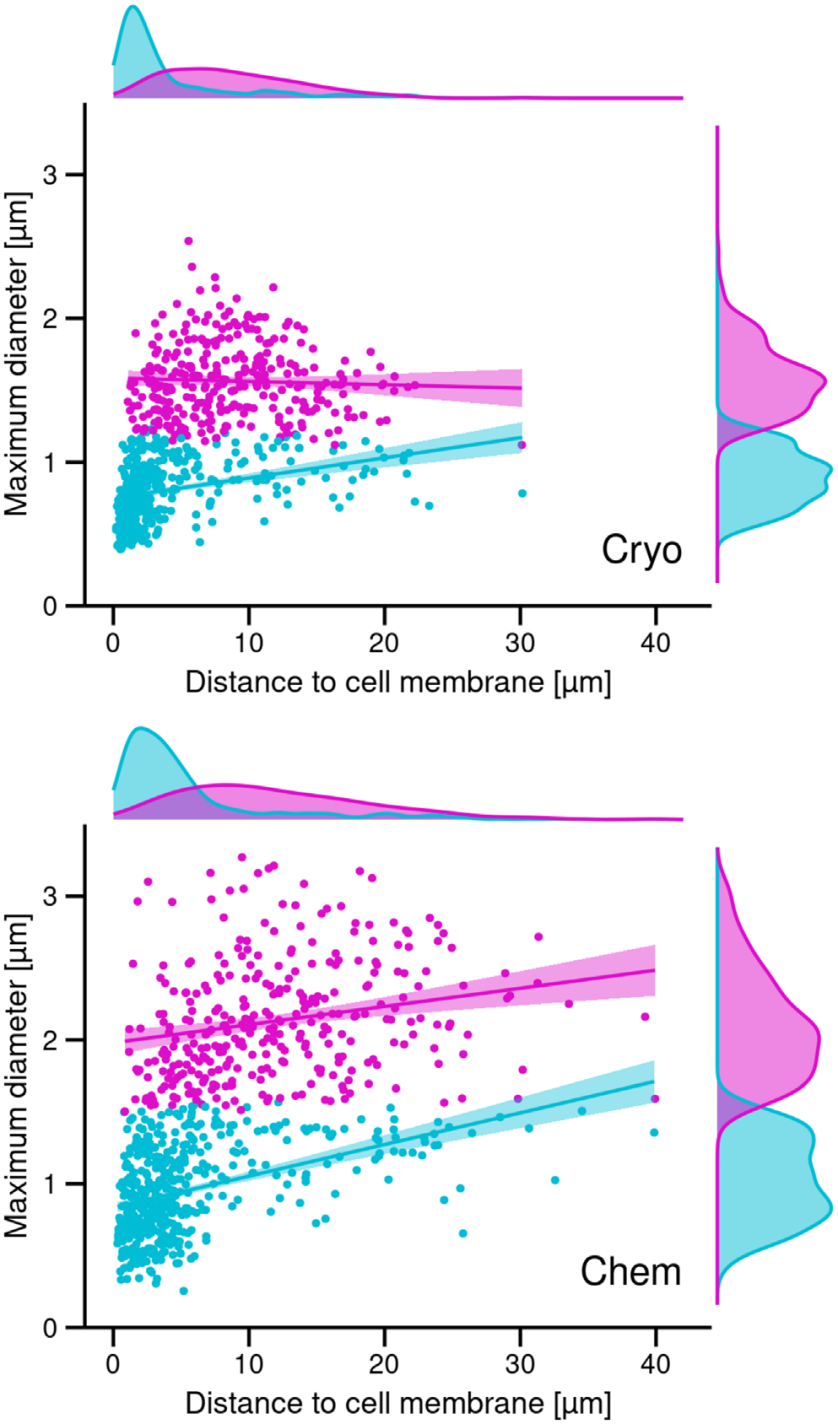
Scatterplots displaying the correlation between the maximum diameter and the distance to the cell membrane in mature lorica‐forming material granules of cryofixed (top) and chemically fixed (bottom) *Schmidingerella* late dividers. The LFM granules were assigned to the large type (magenta) or the small type (cyan), respectively, based on the cluster analyses. The lines with shaded areas represent the linear regression fits with 95% confidence intervals for each cluster. The marginal density plots display the distributions of positions relative to the cell membrane and of the two LFM granule size classes, respectively (cp. Figures S6, S7 and S9).

**FIGURE 6:**
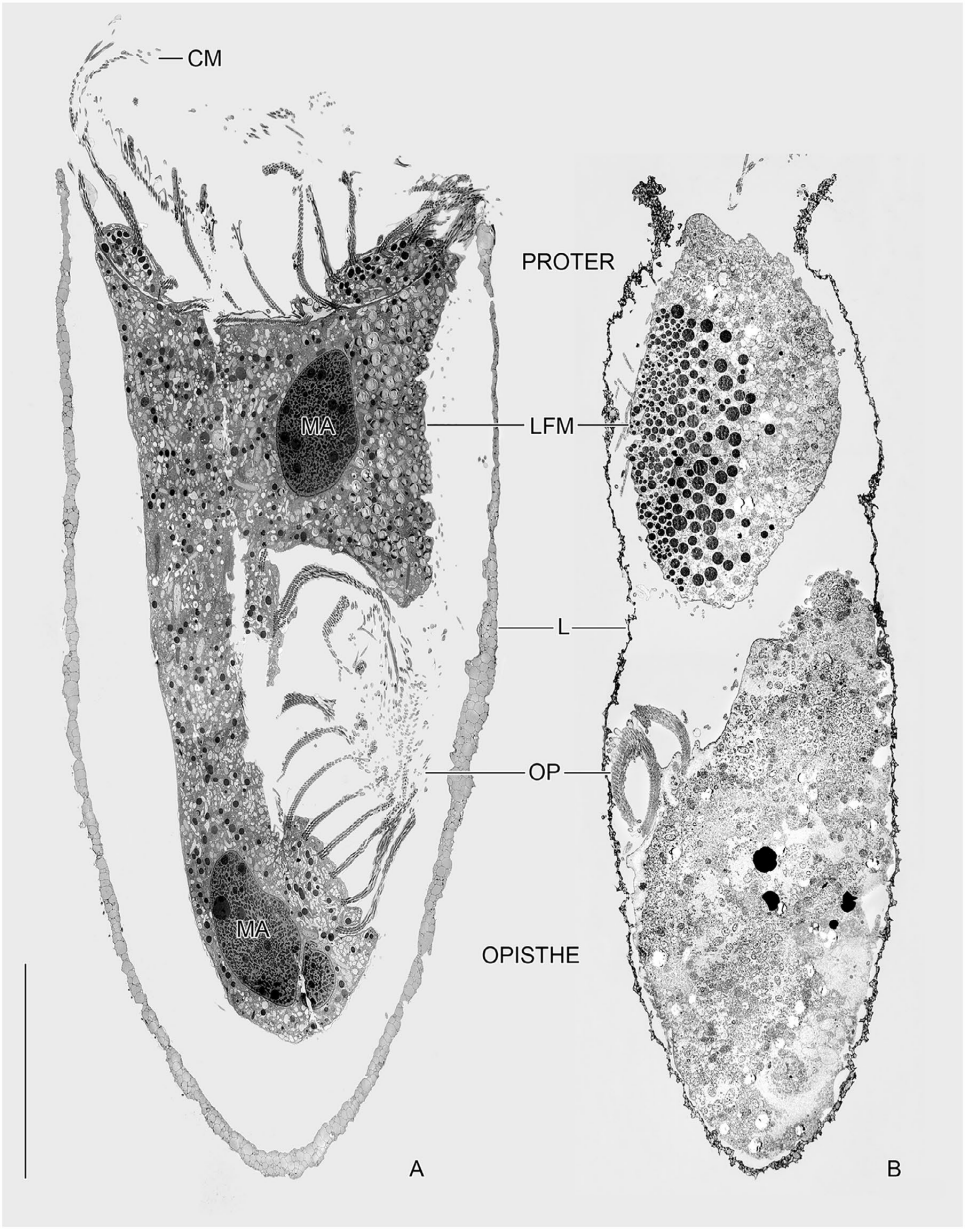
Ultrathin longitudinal sections of entire *Schmidingerella* late dividers after cryofixation (A) and chemical fixation (B) facing their ventral sides. The proter containing the lorica‐forming material and the opisthe are connected by a dorsal cytoplasmic strand (A). The opisthe's oral primordium (future adoral zone of membranelles) has evaginated. Both the proter and opisthe have one macronuclear nodule each (A). These nodules are not identical to those of morphostatic (non‐dividing) specimens but originate from the division of the previously fused nodules with replicated genetic material (Agatha et al. 2025). The superior quality of the cryofixation is obvious, resulting in a much better preservation of the cytoplasm and the membranes. CM, collar membranelles; L, lorica; LFM, lorica‐forming material; MA, macronuclear nodules; OP, oral primordium. Scale bar 30 μm.

In both the cryofixed and chemically fixed very late dividers, the mature granules cluster just underneath the cell cortex of the lateral ciliary field and are ready for secretion (Figures 2F, 4E,F, 5, 6 and S6, S7, S8, S9). Since distinct cytoskeletal structures or predefined pores mediating the secretion of the LFM granules are apparently lacking, the process seems to be a commonly regulated exocytotic event restricted to the region of the cluster. Here, the cell cortex possesses small and irregularly arranged alveoli (flattened unit membrane vesicles) (Figure 4E,F). Four longitudinal sections of a cryofixed very late divider reveal that the LFM cluster commences about 15 μm posteriorly to the apex of the peristomial rim and thus posteriorly to the adoral fiber and the nematodesmata of the about 19 collar membranelles (for the ultrastructure of the oral ciliature, see Gruber et al. 2020). The cluster is 40–41.6 μm long in the ultrathin sections of two cryofixed very late dividers, while on average 36.5 μm in four Bouin‐fixed methyl blue eosin‐stained very late dividers studied by Agatha et al. (2025).

In accordance with a previous light microscopical study on stained material (Agatha et al. 2025) and the present live observations (see below), statistical analyses support the unique existence and distinct spatial arrangement of two size classes in mature granules (Figures S5, S6 and S7). The secretion of the small mature granules not only precedes that of the large ones but apparently facilitates/enables the latter process. In cryofixed late dividers, the small mature granules measure 394–1231 nm (809 ± 207 nm), while the large mature granules range from 1120 nm to 2538 nm (1565 ± 259 nm) (Figures S3 and S5; Table S1). In chemically fixed late dividers, the small mature granules range from 254 nm to 1567 nm (957 ± 315 nm), while the large granules measure 1504–3271 nm (2132 ± 406 nm) (Figures S3 and S5; Table S1). Compared to cryofixed late dividers, these granules are significantly larger: by 18% for small granules and 36% for large granules (Figures S3 and S5; Table S2). In cryofixed very late dividers, the morula‐shaped granules overlap in size with the small mature granules, whereas they are on average much smaller than the large mature granules (Figure S3; Tables S1, S2).

The small granules form a narrow longitudinal strip just beneath the cell cortex and the monokinetids' microtubular ribbons of the lateral field (Figure 4E,F), while the large granules are positioned proximally and laterally, embedding the strip (Figures 5 and S6, S7, S8, S9). The still present morula‐shaped stages are primarily located at the cell's center or at the periphery of the cluster (Figures 2F and S8A). The width of the strip with small mature granules could not be determined from TEM sections but was estimated to be approximately 10 μm in the methyl blue‐eosin‐stained very late divider examined by Agatha et al. (2025). Longitudinal sections of cryofixed and chemically fixed very late dividers consistently reveal conspicuous invaginations within the strip of small mature granules, which are absent in the surrounding cell periphery (Figures 2F and 6A, S6, S7, S8). The region with invaginations extends up to 37.5 μm in four cryofixed specimens and approximately 38.9 μm in one chemically fixed specimen, commencing about 16 μm posteriorly to the peristomial rim's apex.

### 3.2 Relative Carbon, Oxygen, and Nitrogen Content of Granules

Electron energy loss spectroscopy was employed for estimating the chemical similarity of the small and large mature granules. In the wide range electron energy loss spectra of the mature granules, distinct peaks were only detected for carbon, nitrogen, and oxygen, which were individually measured near their specific energy loss edges.

In the cryofixed cell, the measured areas (Figure S10A–C) mainly differ in their nitrogen contents (Figure S10E): it is highest in the center of the large mature granule, followed by that of its electron‐dense cap and of a small mature granule; the lorica wall contains the least nitrogen. The differences are less pronounced for oxygen and carbon (Figure S10D,F).

In a chemically fixed late divider, one subunit of a morula‐shaped granule, an electron‐dense mature granule, and the lorica wall were analyzed. The mature granule had a higher nitrogen content than both the subunit of the morula‐shaped granule and the lorica wall. The granules and the lorica wall were similar in their oxygen and carbon contents (data not shown).

### 3.3 Live Observations of Dividers Containing LFM

Live observations on late dividers, the secretion process, and the lorica formation are summarized on figure plates and optionally available as videos in the supporting information (Videos S1–, S6).

**FIGURE 7:**
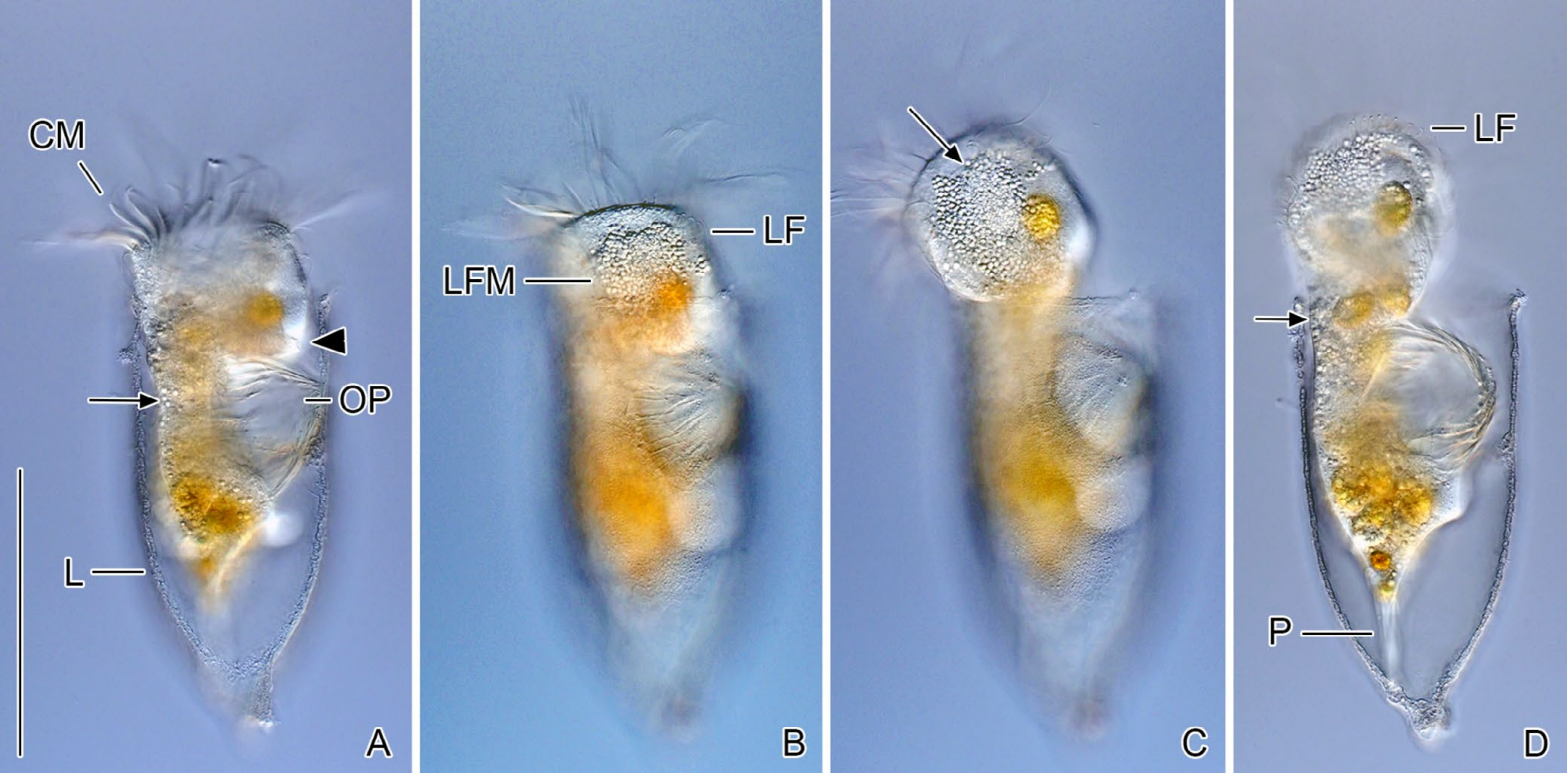
Time series of same *Schmidingerella* very late divider in vivo showing the gradual separation of proter (anterior division product) and opisthe (posterior division product). The cluster of highly refractile mature granules of lorica‐forming material lies just underneath the lateral ciliary field (B). Arrowhead marks the proter's future peduncle (A). Arrow in (C) denotes the longitudinal strip of small granules embedded in large granules, while arrows in (A, D) mark the dorsal cytoplasmic strand connecting proter and opisthe. CM, collar membranelles; L, lorica; LF, left ciliary field with elongated anterior cilia; LFM, lorica‐forming material; OP, oral primordium; P, contractile peduncle of the opisthe attached to the lorica bottom. Scale bar 100 μm.

Non‐dividing (morphostatic) *Schmidingerella* specimens swim in a relatively straight path with a slow rotation about their main cell axis, occasionally interrupted by abrupt stops and changes in swimming direction. Since they have a champagne flute‐shaped lorica, which is transparent in the light microscope, the cell, its somatic ciliature, some of its internal structures, and the developing oral primordium are clearly visible within the lorica (Figure 7A–D).

The LFM granules become perceptible as refractile granules in middle dividers under the light microscope. Initially scattered throughout the cell, they soon begin accumulating around the developing oral primordium (Agatha et al. 2025). This clustering of highly refractile LFM is a striking process, readily observable even under the stereo microscope, using dark field illumination. In late dividers, characterized by the circular arrangement of the opisthe's adoral membranelles in a ventral subsurface pouch, the conspicuous cluster of LFM granules is clearly visible anterior to the oral primordium and beneath the proter's long, densely ciliated lateral ciliary field (Figure 7A–D). Even in live specimens, small and large LFM granules (about 500 nm vs. 1–2 μm) can be distinguished under the light microscope (Figure 7C).

The LFM granules undergo a size‐based sorting in the highly fluid cytoplasm of very late dividers: small granules push through the large ones, eventually forming a longitudinal strip just underneath the more loosely ciliated left portion of the lateral ciliary field (Figure 7C). In some specimens, the cell membrane appears indented, possibly indicating a surface expansion through the integration of additional membrane material (Figure S8). Posterior to the refractile LFM cluster, a distinct oblique ventral notch, the future division furrow, becomes apparent in very late dividers, while the proter and opisthe are connected by a dorsal cytoplasmic strand (Figure 7A,D). Although the opisthe already has an evaginated and obliquely orientated membranellar zone covering the posterior surface of the ventral notch, the swimming behavior remains similar to earlier division stages. This is likely because the proter's membranelles are much more active than those of the opisthe, which are mostly bent towards the peristomial field and covered by the lorica.

The opisthe's oral ciliature is completed by cytoplasmic extensions (tentaculoids and striae) longitudinally extending between and on the membranelles, respectively; both extensions usually contain ejectable capsules and mucocysts (Figure S1B).

As the dorsal cytoplasmic strand narrows over the course of a few minutes, the opisthe's oral primordium reorients nearly perpendicular to the main cell axis, its membranelles begin moving more vigorously, and the peristomial field initiates pumping movements.

Very late dividers swim in narrow spirals, frequently with back‐and‐forth movements, seemingly aiding the separation of proter and opisthe. Just before cell division, the proter extends far beyond the lorica rim, orienting itself at an oblique to perpendicular angle relative to the opisthe's main cell axis (Figure 7C,D). At this advanced stage of division, the proter has a posteriorly broadly rounded bulge in the ventrolateral cell portion, likely corresponding to the lateral lobe sensu Campbell (1926, 1927), which bears the extremely long lateral ciliary field (Agatha et al. 2025). The elongated anteriormost cilia of the right and left ciliary fields extend posteriorly, running parallel to the cell surface and moving only in their distal portions, while the remaining cilia of both fields beat vigorously in both the proter and opisthe. The macronucleus, positioned in the proter's left cell half, usually divides after separation from the opisthe. Immediately after fission, the proter starts LFM secretion.

### 3.4 Secretion and Behavior of LFM

**FIGURE 8:**
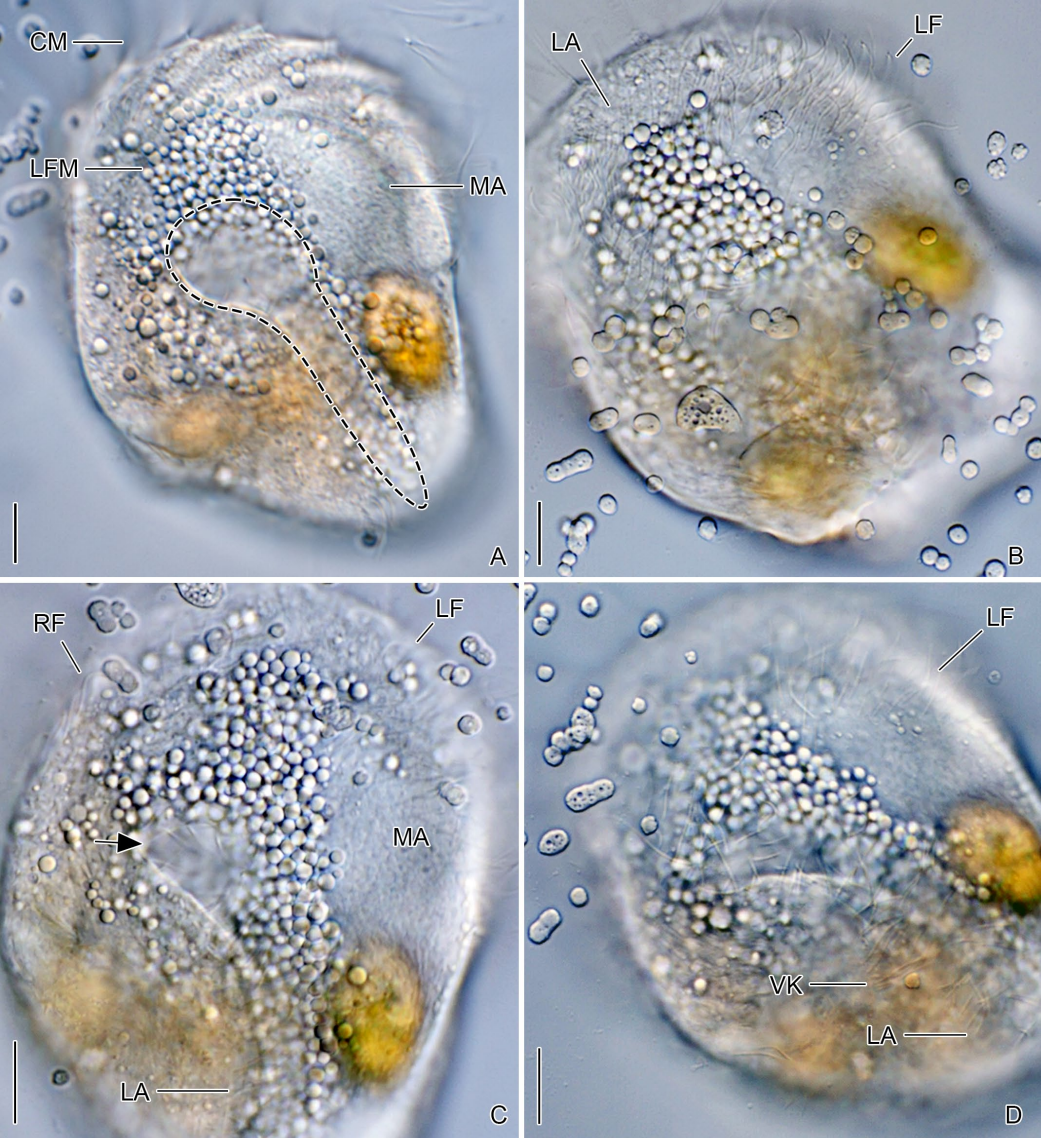
Interference contrast micrographs showing a living *Schmidingerella* proter which originated from the division of the very late divider shown in Figure 7. The granule cluster of lorica‐forming material is underneath the lateral ciliary field (A‐D). Upon a massive release of the material, a longitudinal furrow and a cavity (A: dashed line, C: arrow) form. CM, collar membranelles; LA, lateral ciliary field; LF, left ciliary field; LFM, lorica‐forming material; MA, macronuclear nodules; RF, right ciliary field; VK, ventral kinety. Scale bars 5 μm.

Congruent with our ultrastructural findings, the light microscopic observations on proters after division (postdividers) could not reveal a predefined pore or gutter for LFM secretion (Figure 8). Upon an unknown, probably intrinsic stimulus, initially, the small mature granules are extruded from the longitudinal strip underneath the left portion of the lateral ciliary field. Next, the large mature granules are progressively released exclusively in the region of the lateral ciliary field. The secretion of LFM causes the development of an inclined obclavate furrow (Figures 8A,C and S11A). Its anterior portion is deepened, forming a pre‐equatorial cavity (Figure 8A,C). A sigmoidal bulge delimiting the furrow on the right‐hand bears three or four ciliary rows commencing just posteriorly to the collar membranelles and extending to the posterior cell end. These kineties comprise the ventral kinety and two or three ciliary rows of the adjacent lateral field (Figures 8D and S11A). The remaining lateral kineties extend through the furrow and cavity to the rear end (Figure 8C,D). The left margin of the furrow is distinct only in the posterior cell half, where large mature granules are lined up in both furrow edges.

**FIGURE 9:**
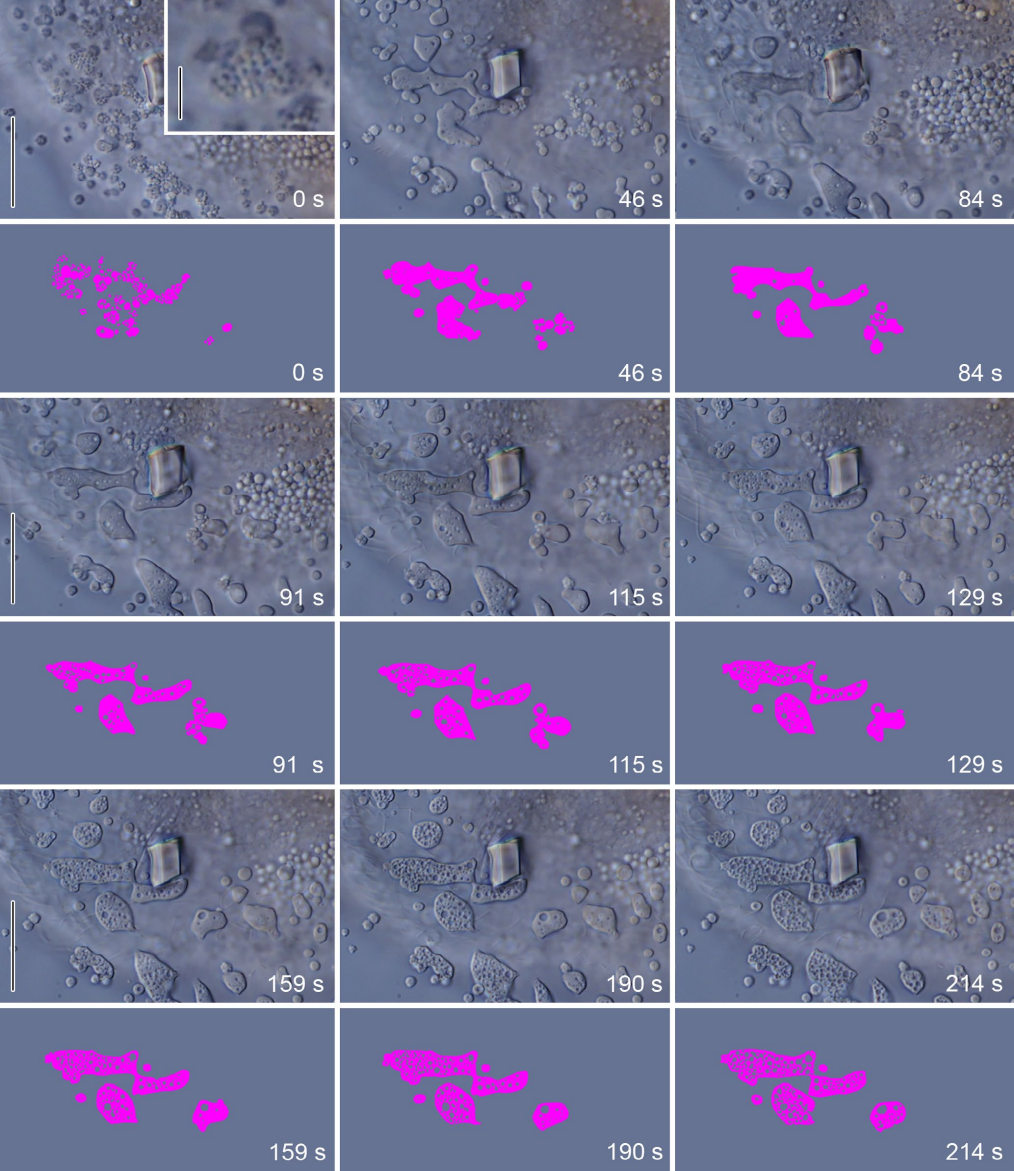
Timeline of interference contrast micrographs showing the behavior of lorica‐forming material (upper rows) secreted by a *Schmidingerella* proter and the identical material clumps marked by magenta (lower rows) (cp. Video S5). Interestingly, only fused granules distinctly expand by obtaining a foamy structure with minute alveoli (chambers) that likely correspond to those of the finished, hard lorica wall. Magnified inset (first image) shows just released granules disintegrated into globular subunits. Scale bars 10 μm, insert 2 μm.

Upon contact with the seawater medium, the initially homogeneous granules instantly undergo a structural reorganization, occasionally transforming from a globular structure into an aggregation of numerous smaller subunits, resembling the morula‐shaped stage (Figure 9, 0 s). These subunits swiftly swell and merge with adjacent granules to form an amorphous mass (Figure 9, 46 s). The densely arranged lateral cilia move vigorously, generating an LFM flow towards the posterior cell end. Thereby, they facilitate the contact between the granules and their subsequent mutual cohesion. The remaining somatic ciliature appears largely uninvolved in LFM transport. After merging, the granules undergo a progressive transformation as minute alveoli (chambers) form and gradually expand, creating an increasingly foamy structure resembling the finished lorica wall (Figure 9). In specimens unable to freely move, the LFM secretion might commence even prior to fission, resulting in the formation of an alveolar, ring‐shaped structure, or it might be delayed (not immediately after separation from the opisthe). Such rings or short tubes (malformed loricae) were also formed by specimens in culture wells under the stereo microscope using the integrated illumination. These malformed loricae are neither found in field material nor in the focus of the present study.

### 3.5 Lorica Formation

Typically shaped loricae dominated in the culture material, but their formation was only partly observed in undisturbed, freely swimming specimens under a stereo microscope with external illumination. Consequently, the overall process could be documented, while finer details remained unclear due to the limited optical resolution and depth of focus.

**FIGURE 10:**
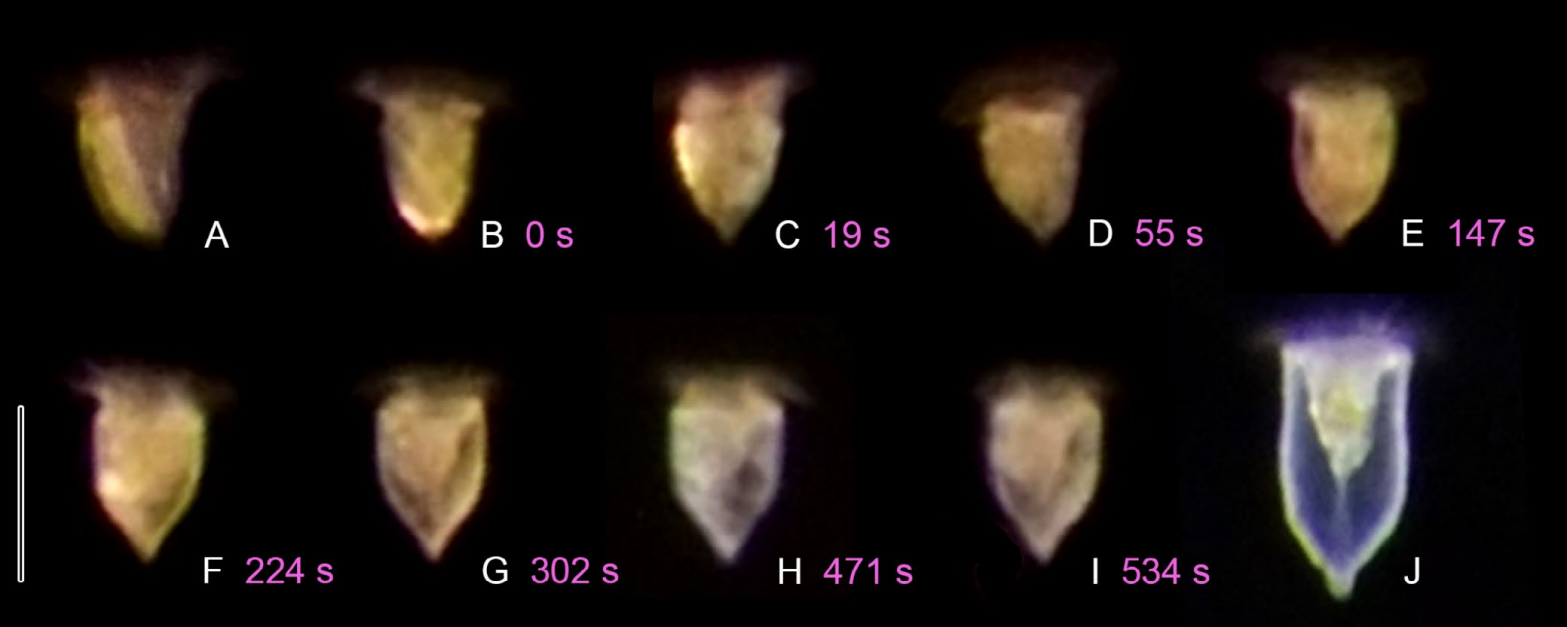
Snapshots of lorica formation in two freely moving *Schmidingerella* proters and a morphostatic (non‐dividing) specimen with a finished lorica captured under a stereo microscope with up to 115× magnification and an external LED light source (cp. Video S6). (A) Proter 1 with a distinct oblique whitish strip of lorica‐forming material. (B–I) Proter 2 in sequential stages of lorica formation: First, the pointed end of the posterior lorica process becomes recognizable (B–D). Then, the lorica wall is discernible (E) and the typical lorica shape with a distinct posterior process merging into a cylindroidal portion develops (F). During this stage, the proter is markedly smaller and now attached to the lorica bottom by its peduncle (G). The lorica wall seemingly becomes thicker (H); at this timepoint, the proter aborted lorica construction (I). Thus, complete lorica formation including an anterior extension was not observed. (J) Morphostatic specimen with a typically completed lorica which is translucent but highly refractile under dark field illumination. The numbers give the timepoints in seconds during the continuous observation of Proter 2. Scale bar 100 μm.

The proter enlarges, potentially by the uptake of water, obtaining an ellipsoidal shape just prior to or after the cytoplasmic strand is cut. Afterward, it swims slightly faster than the very late divider, moving in wide spirals occasionally interrupted by back‐and‐forth motions combined with changes in swimming direction. The remains of the cytoplasmic strand previously linking proter and opisthe are usually resorbed within a minute. Concurrently, the LFM cluster, which appears as a highly refractile, oblique whitish strip, also stretches, extending along the entire length of the cell (Figure 10A). Within the first two minutes after separation, a considerable amount of LFM is secreted, generating a thin cover on the ellipsoidal cell body posterior to the membranellar zone, initially recognizable only by the sparkling appearance of the cell surface. Simultaneously, the narrowed posterior lorica process begins to form, first appearing as a pointed end of the incomplete thimble‐shaped lorica (Figure 10C,D). The lorica wall becomes partially discernible as the cell achieves a somewhat asymmetric shape (Figure 10E,F). Parallel to the gradual elongation of the posterior process, the cell seemingly reduces its volume by water loss and extends its previously invisible peduncle. Thereby, it detaches from the lorica, which is now about two‐thirds of the final lorica length (Figure 10G). Over time, the newly formed lorica wall becomes more refractile and increasingly visible, possibly due to a continuous swelling. Meanwhile, the proter's peduncle continues to extend, and the cell progressively adopts an elongate obconical shape, similar to that of morphostatic specimens (Figure 10H–J). At this stage, the cell still contains a visible amount of LFM. The process delineated here was observed twice, though we could not follow the lorica completion, specifically its subsequent anterior elongation by about one‐third of the final lorica length and the addition of a minute spiraled collar.

The fully formed typical lorica in the investigated strain measures approximately 200 μm in length and has an opening diameter of about 80 μm (Figure 10J). Its anterior portion is cylindroidal and characterized by a ring‐shaped subapical bulge and a thin, spiraled collar up to 5 μm long. The posterior portion is broadly rounded, merging into a bluntly pointed, hollow process approximately 30 μm long and 15 μm wide at its base (Figure S12A,B). The lorica wall is about 2 μm thick, usually perforated by minute pores (about 0.3 μm across), colorless, and composed of alveoli of variable sizes (0.5–2 μm; Figure S12C,D). The outer surface exhibits a reticulate pattern of ridges, while the inner surface is smooth (Agatha and Bartel 2022).

## 4 Discussion

### 4.1 LFM Maturation

**FIGURE 11:**
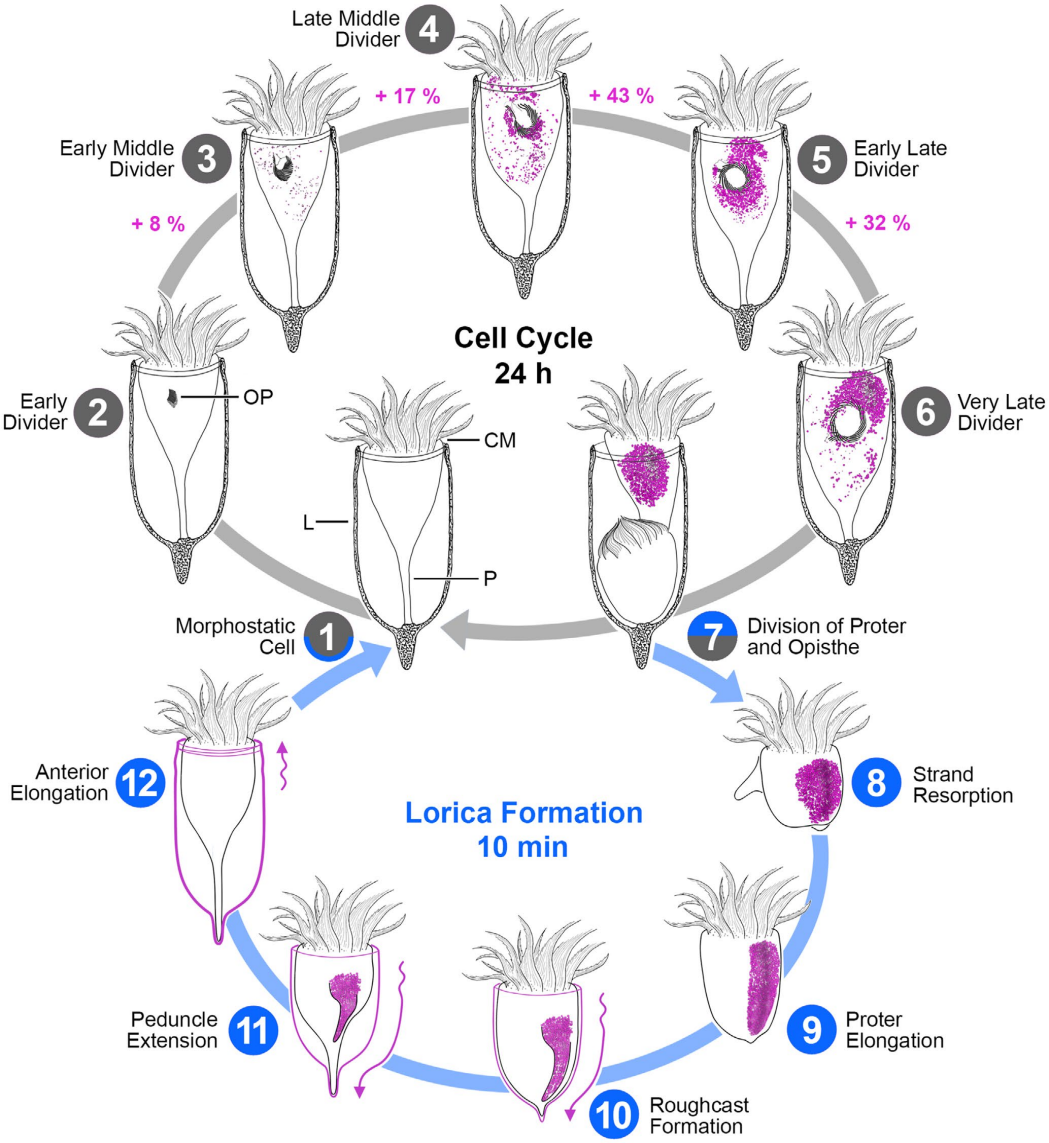
Scheme of the daily cell cycle in *Schmidingerella* highlighting lorica‐forming material (LFM; magenta) production (adapted from Agatha et al. 2025) and rapid lorica formation by the proter. The upper cycle depicts ventral views of the morphostatic specimen (1) and five division stages (2–6) characterized by the development of the oral primordium (OP). LFM progressively accumulates (percentages of final average volume) and clusters in the proter (3–7). Following transverse division (7), the opisthe retains the parental lorica (L). The proter, however, constructs a new lorica while steadily swimming by means of the collar membranelles (CM) in spirals and rotation about its main cell axis. The process begins with the resorption of the previous cytoplasmic connection with the opisthe (8) and the proter's elongation, obtaining an ellipsoidal shape (9). The secreted LFM initially forms a layer on the cell surface, coinciding with the emergence of a ventral furrow (10). As the peduncle (P) extends, the expanding lorica wall becomes visible (11). Finally, the proter elongates the lorica anteriorly (12) and the alveolar lorica wall hardens (1).

Tintinnids are capable of dividing daily, forming a new lorica each time (Figure 11; this study; Brownlee 1982; Laval‐Peuto 1981). During division in *Schmidingerella*, the LFM is first detected as scattered granules in middle dividers by light microscopy (Agatha et al. 2025). In the subsequent division stages, additional LFM granules accumulate, and all granules are translocated into the anterior ventral cell portion, the proter, clustering underneath its long lateral ciliary field (Campbell 1926, 1927; Agatha et al. 2025). Just before cell division, the LFM occupies on average 7% of the cell volume in *Schmidingerella* (up to 21,860 μm^3^), seemingly arranged in a peripheral strip of small granules embedded in large granules (Agatha et al. 2025). Given this high production rate of LFM, one would expect an extraordinarily voluminous rough endoplasmic reticulum (rER) with stacks of narrow cisternae comparable to those found in other organisms with high secretion demands (luminal spacing about 50 nm in mammals and 30 nm in yeast; Schwarz and Blower 2016). Moreover, stacked Golgi bodies should facilitate the extensive biosynthetic and membrane trafficking processes (More et al. 2020).

Surprisingly, our cryofixed monoclonal *Schmidingerella* cells exhibit significantly fewer and smaller Golgi bodies along with a sparsely developed rER compared to the ciliate *Tetrahymena*, which does not form a shell (Franke and Eckert 1971; Franke et al. 1971), or other shell‐forming protists (e.g., Anderson 1988; Harrison et al. 1976). In our specimens as well as in the few other tintinnids studied to date, the rER is largely confined to the vicinity of the macronuclear envelope and the mitochondria (this study; Hedin 1975; Wasik and Mikołajczyk 1992). In contrast, the cytoplasm contains innumerable free ribosomes (this study; Laval‐Peuto 1975; Sokolova and Gerassimova 1984) and relatively large, irregular smooth vesicles with fluffy or granular content. In *Schmidingerella*, such vesicles are frequently found around and interspersed among those already containing clearly identifiable LFM granules (Figures 1, 3B,D and S1A, S4A). Based on our ultrastructural data, the vesicles' role in LFM genesis and maturation remains unverified.

Previous studies on chemically fixed tintinnids assumed that the site of LFM production is the “vesicular reticulum” which opens into the (pericellular) space between the cell membrane and the perilemma, an additional membranous sheet enclosing the cell in Perilemmaphora (Laval‐Peuto 1975, 1994; Laval‐Peuto et al. 1979; Sokolova and Gerassimova 1984). Our cryofixed material of unprecedented quality clearly displays that this structure is an artifact.

We hypothesize that LFM production does not follow the conventional, regulated secretion pathway through the rER and Golgi apparatus as in other ciliates (Richardson and Dacks 2022). It is likely that specialized mechanisms facilitate the formation of granules in secretory vesicles of *Schmidingerella*, as large amounts of LFM must be efficiently generated, concentrated, and transported. Thus, the precise organellar origin and the initiation of this process remain unclear.

The earliest structures identifiable as LFM are membrane‐bound, loosely packed electron‐dense morula‐shaped granules (this study; Wasik 1998; Wasik and Mikołajczyk 1992). These unique granules have been reported exclusively from tintinnids, namely the genera *Cymatocylis* (Wasik and Mikołajczyk 1992), *Cyttarocylis* (Laval‐Peuto 1975), *Parafavella* (Sokolova and Gerassimova 1984), and *Petalotricha* (Laval 1972; Laval‐Peuto 1975).

Three additional granule types are interpreted as consecutive stages of maturation following the morula‐shaped one in *Schmidingerella* dividers: (1) compact morula‐shaped granules, (2) inverse morula‐shaped granules, and (3) mature granules in two size classes. We assume a distinct swelling of the granules during the presumed transformation to the mature stage, as evidence for a vesicle growth due to the fusion of existing granules could not be detected in the ultrathin sections. The mature stages differ in their electron densities depending on the fixation method. In cryofixed specimens, they appear largely electron transparent with amorphous content, except for their electron‐dense surface and caps. In contrast, a pre‐fixation with glutaraldehyde and a post‐fixation with high concentrations of osmium tetroxide result in an extreme electron density of the entire granules. Compared to their cryofixed counterparts, the chemically fixed granules are larger (Tables S1 and S2) likely due to the effects of the double fixation (Tzeng et al. 1981). Notably, the inverse granule type representing the transition stage between morula‐shaped and mature granules, which supports our hypothesis on LFM maturation, was detected exclusively in cryofixed specimens.

The clustering of mature granules underneath the proter's lateral ciliary field (lateral lobe sensu Campbell 1926) was already noted in the historical literature. However, the occurrence of small and large mature granules and the formation of a peripheral strip of small granules embedded in large ones was only recently discovered in stained material of *Schmidingerella* species (Agatha et al. 2025). These findings are now confirmed by ultrastructural data and live observations (Figures 2F, 5, 6 and S5, S9). Campbell (1927) hypothesized the presence of two chemically distinct types of LFM in *Schmidingerella serrata* (reported as *Favella serrata* ): one forming the alveolar walls and another comprising their content. We infer a single transformation pathway for both granule size classes from the same staining properties after methyl blue‐eosin and protargol application (Agatha et al. 2025), their identical ultrastructure, the highly similar relative content of carbon, oxygen, and nitrogen, and the uniform lorica wall texture. Additionally, evidence for division/budding of large granules or fusion of small ones is missing. The comparatively low abundance of morula‐shaped granules, and particularly the intermediate stages, suggests a rapid transformation to mature granules. Thus, the present study is the first to provide a near‐natural snapshot of these subcellular processes in a tintinnid excellently documented in our cryofixed specimens.

### 4.2 LFM Secretion Induced Morphological Changes

So far, cell division in a living tintinnid has only rarely been described (Biernacka 1952; Campbell 1926, 1927; Laval‐Peuto 1981, 1994). The findings largely match our observations in *Schmidingerella*, for which we provide the first detailed documentation by images and videos.

LFM secretion starts immediately after cell division (this study; Biernacka 1952; Laval‐Peuto 1981). The proter remains highly motile during the entire secretion process, actively swimming when undisturbed (this study; Biernacka 1952; Laval‐Peuto 1981). In rare cases, granules are extruded while proter and opisthe are still connected, leading to the formation of rings or other malformed loricae (Kofoid 1930; Schweyer 1909).

Several LFM secretion sites have been proposed in the historical literature, including the entire cell surface (“peeling”; Busch 1925; Entz Jr. 1909), the “lateral lobe” or bulge (Campbell 1926; Hofker 1931), the cell mouth (Campbell 1927; Kofoid 1930), the cytopyge (cell anus; Campbell 1927), a region near the adoral zone of membranelles (often called the “peristome”; Biernacka 1965; Schweyer 1909), and a pore or thin gutter posterior to the membranellar zone, near the center of the LFM cluster (Laval‐Peuto 1981). We provide evidence for *Schmidingerella* that secretion occurs in a restricted area, namely, exclusively through a longitudinal strip in the lateral ciliary field (this study; Brownlee 1982). In several other tintinnid species with a lateral ciliary field and a similar cell morphology (*Eutintinnus*, *Favella*, *Stenosemella*, *Tintinnopsis*), a comparable LFM accumulation anterior to the developing oral primordium and secretion in the region of the ventral cluster were reported (Agatha et al. 2013; Biernacka 1952; Brownlee 1982; Campbell 1926, 1927; Fauré‐Fremiet 1924). Yet, these limited data do not allow a generalization as taxa with different cell morphologies (e.g., members of the genera *Amphorellopsis*, *Amphorides*, *Bursaopsis*, *Salpingacantha*, *Salpingella*, and *Steenstrupiella*; Agatha and Strüder‐Kypke 2014) and/or different somatic ciliary patterns (e.g., *Antetintinnidium* and *Tintinnidium*; Ganser and Agatha 2019) might show deviations regarding LFM generation, accumulation, and secretion. Nevertheless, the LFM needs to be transported into the anterior cell portion prior to division, enabling the proter to immediately form a new lorica.

The densely arranged cilia and ciliary rows of the lateral field plus the unique network of basal body associated microtubular ribbons (Figure 4E,F; Agatha et al. 2022; Gruber et al. 2018) raise the question of how the vast number of LFM granules pass the cell cortex for secretion. Our live observations revealed a two‐tiered secretion process: first, the small granules are released, followed by a substantial secretion of large granules. We suggest that the exocytosis of the small granules locally expands the membrane, widening the microtubular network associated with the lateral ciliary field and thereby facilitating the passage of the large granules. The rapid release of intracellular LFM and the resulting volume reduction in this cell region might be responsible for the distinct changes in the proter's morphology, namely, the formation of a longitudinal furrow and cavity in the lateral ciliary field. From these indentations, the remaining granules are secreted.

In summary, our study provides important new information on the site and mechanism of LFM secretion in *Schmidingerella* and provides a basis for inferences about this process in taxa with similar ciliary patterns and cell morphologies. The secretion process follows an unknown stimulus and proceeds in a structured, stepwise manner, that is, the small granules are proposed to facilitate the subsequent release of the large granules in the region of the lateral ciliary field, which is distinctly elongated just after cell division. This discovery, supported by live imaging and ultrastructural analyses, provides new insights into the subcellular dynamics of this crucial secretion process in tintinnid ciliates.

### 4.3 Swelling and Alveolarization of Released LFM

The LFM secreted by tintinnids is frequently described as viscous and slightly sticky (this study; Campbell 1926; Entz Sr. 1885; Hofker 1931; Schweyer 1909). Consistently, a swelling of the LFM granules has been reported, which immediately begin to merge with each other (colloidal aggregation; Campbell 1927) and progressively harden (this study; Campbell 1926, 1927; Hofker 1931; Kofoid 1930; Laval‐Peuto 1981; Schweyer 1909). This gradual fusion of LFM granules has also been documented by scanning electron micrographs in *Favella* and *Schmidingerella* (Figure S11B,C; Agatha et al. 2013).

In taxa with an alveolar lorica wall texture, fused granules generally swell distinctly and generate an alveolar or foamy texture (this study; Campbell 1926, 1927; Kofoid 1930; Laval‐Peuto 1981; Schweyer 1909). In disturbed specimens, however, some granules remain isolated, form only a minute chamber, and thus display a merely limited swelling (Figure 9). We hypothesize that seawater infiltrates the alveoli during the swelling process and is eventually enclosed by the hardened wall material. This aligns with the ultrastructural study of the finished lorica wall in *Schmidingerella*, which consists of a trilaminar structure, that is, one alveolar layer enclosed by compact inner and outer layers, previously suggested to be formed in a single secretion cycle of uniform material (Agatha and Bartel 2022).

### 4.4 Live Observations Unveil Key Stages of Lorica Formation

Live observations of lorica formation are inherently time‐consuming and challenging due to the proter's rapid movements (this study; Biernacka 1952) and high sensitivity to physiological, chemical, or mechanical stress (this study; Laval‐Peuto 1981; Schweyer 1909). Most importantly, cells must be suspended in a sufficient volume of water to move freely during and after division. These constraints limit the level of detail that can be documented through image and video capturing. Nevertheless, we were able to observe key stages of lorica formation in five postdividers during this process, allowing for some generalizations.

The new lorica is exclusively formed by the freely swimming proter, as the regular LFM secretion only starts after its separation from the opisthe and the abandoning of the parental shell (this study; Laval‐Peuto 1981). Thus, a collaboration between proter and opisthe, as postulated by Campbell (1926, 1927) and Kofoid (1930) for constructing the highly elaborate posterior lorica process or other lorica structures, does not take place. Our observations on *Schmidingerella* largely match those of Laval‐Peuto (1981). For the first time, we document the emergence of a longitudinal furrow within the densely ciliated lateral field following the secretion of a considerable amount of LFM.

A two‐phase lorica construction as in *Schmidingerella* has also been suggested in other tintinnid taxa (Bernatzky et al. 1981; Biernacka 1952, 1965; Entz Sr. 1885; Laval‐Peuto 1981). The first phase of lorica formation is remarkably rapid, lasting only a few minutes in *Schmidingerella* and *Favella* (this study; Laval‐Peuto 1981). In our *Schmidingerella* specimens, unfortunately, we could not trace the second phase of lorica construction, namely, the anterior elongation with the final addition of the small collar composed of minute alveoli. Likewise, the generation of loricae with partially or entirely spiraled walls assigned to the *decipiens* or *coxliella* forms was not examined in our material, although the *Coxliella* morphotype occasionally occurred in our culture. Based on Laval‐Peuto's (1981) observations on *Favella*, we assume that *Schmidingerella* also constructs this lorica form by a spiraled, anteriorward LFM deposition.

The duration of the complete lorica construction varies among tintinnids, lasting approximately 10 min at a water temperature of 18°C in *Favella* (Laval‐Peuto 1981) and *Schmidingerella* (this study), only a few minutes in *Tintinnidium fluviatile* (temperature not mentioned; Entz Sr. 1885), but extending to 4–5 h at about 18°C in *Tintinnopsis* (Biernacka 1952).

We confirm and refine the assumption by Laval‐Peuto (1981) that solely the somatic ciliature is responsible for the lorica formation. Consequently, we exclude an involvement of the proter's adoral membranelles and propose that the LFM is mainly handled by the lateral cilia, which definitely perform the posteriorward LFM flux during the first phase of lorica construction, potentially together with the ventral kinety (this study; Biernacka 1952, 1965; Laval‐Peuto 1981; Schweyer 1909), corresponding to the “ciliary membrane” described by Campbell (1926). Interestingly, Campbell (1927) did not identify a lateral ciliary field in *Schmidingerella serrata* (reported as *Favella serrata* ) and instead proposed a major contribution by the elongated anterior cilia of the right and left ciliary fields, referred to as paroral cilia. According to our detailed live observations, however, LFM accumulates to thick ring‐shaped structures posteriorly to the insertions of these cilia exclusively in specimens affected by suboptimal conditions during lorica formation. A deposition of spiraled LFM ribbons, as mentioned by Laval‐Peuto (1981), cannot be inferred from our live observations on postdividers, but abandoned structures formed like a paper streamer occurred in the sediment of our multiwell plates. No form of lorica repair was detected, as proposed/observed by Biernacka (1965).

We hypothesize that the ciliary movement facilitates the fusion of the extruded granules, which immediately become foamy and swell. The generation of the lorica probably necessitates the interplay of further factors. The ciliary movement and the tintinnid's swimming behavior, together with adhesion forces and a continuous fluid motion, spread the LFM of a certain viscosity to a very thin lorica wall layer on the cell surface. An involvement of the perilemma, a thin sheath covering the cell cortex, could not be detected. The fascinating self‐assembly process yielding the highly elaborated loricae is thus successful only when particular physical, chemical, physiological, and behavioral factors convene.

## 5 Conclusion and Outlook

In the present study on a monoclonal *Schmidingerella* strain from the Northeast Pacific, some gaps in the knowledge about the maturation of the presumably proteinaceous LFM, its secretion, and the lorica construction could be filled, using the unprecedented quality of cryofixed, ultrathin sectioned material. The data demonstrate that (1) the striking morula‐shaped granules represent precursors of the mature granules, (2) the LFM is released in the region of the long lateral ciliary field, and (3) the granules fuse and become foamy, causing a distinct swelling of the material as indicated by a recent volumetric analysis (Agatha et al. 2025).

Our findings are from *Schmidingerella*, which has a hyaline, alveolar lorica with reticulate surface ridges and minute pores, while analyses of LFM maturation, secretion, and lorica formation in taxa with different wall textures and structures are pending. The lorica wall texture (monolaminar, bilaminar, trilaminar, alveolar, compact) and structure (hyaline, agglutinated) are determined by its chemical composition (Agatha et al. 2013) which might also influence the ultrastructure and maturation process of the LFM granules. More data are thus required to fully understand the evolution of lorica formation in tintinnids, elucidating the complex synergy of LFM biosynthesis, secretory mechanisms, cell behavior, and selective environmental factors.

## Author Contributions

S.A. and M.H.G. conceived and guided the study. M.H.G. and B.W. accomplished the cultivation of the *Schmidingerella* strains and food algae. B.W. performed the transmission electron microscopy, that is, thin‐sectioning, TEM imaging, as well as analyzing and processing the TEM images. Additionally, B.W. wrote the corresponding methods sections. S.A. conducted the chemical fixation and processed the digital images and edited the videos. M.H.G. and S.A. performed the cryofixation and live investigations, namely, image and video capturing and their analyses. M.H.G. performed the data analyses in R and generated the plots. S.A. and M.H.G. wrote the manuscript. All authors read and approved the final manuscript.

## Conflicts of Interest

The authors declare no conflicts of interest.

## Supporting information


**Table S1.** Morphometrics of lorica‐forming material granules in cryofixed and chemically fixed *Schmidingerella* dividers.
**Table S2.** TukeyHSD post hoc test results on lorica‐forming material granules linked with Figure S3.
**Figure S1.** Ultrathin sections of cryofixed and chemically fixed *Schmidingerella* late dividers showing the co‐occurrence of different lorica‐forming material granule stages.
**Figure S2.** Scatterplot and boxplots showing the relationship between the maximum diameter and the number of subunits in morula‐shaped lorica‐forming material granules of cryofixed and chemically fixed *Schmidingerella* late dividers.
**Figure S3.** Box plots of the maximum diameters for three lorica‐forming material granule types (morula‐shaped, small mature, large mature) in cryofixed and chemically fixed *Schmidingerella* late dividers.
**Figure S4.** Ultrathin sections of compact morula‐shaped lorica‐forming material granules in cryofixed and chemically fixed *Schmidingerella* late dividers.
**Figure S5.** Histograms and cluster analyses of mature lorica‐forming material granule dimensions in cryofixed and chemically fixed *Schmidingerella* late dividers.
**Figure S6.** Series of three longitudinal ultrathin sections through the cluster of mature lorica‐forming material granules in a cryofixed *Schmidingerella* late divider from the cluster’s center to its margin.
**Figure S7.** Series of three longitudinal ultrathin sections through the cluster of mature lorica‐forming material granules in a chemically fixed *Schmidingerella* late divider from the cluster’s centre to its margin.
**Figure S8.** Ultrathin longitudinal sections showing the arrangement of the mature lorica‐forming material granules in cryofixed and chemically fixed *Schmidingerella* late dividers.
**Figure S9.** Analyses of two series each comprising three longitudinal ultrathin sections through the cluster of mature lorica‐forming material granules in a cryofixed and a chemically fixed *Schmidingerella* late divider, respectively.
**Figure S10.** Electron energy loss spectroscopy (EELS) of lorica‐forming material granules and the lorica wall in a cryofixed *Schmidingerella* late divider.
**Figure S11.** Scanning electron micrographs of *Schmidingerella* specimens from a different strain previously cultured in our lab and a *Favella* specimen.
**Figure S12.** Ultrathin sections of finished cryofixed loricae of *Schmidingerella*.

**Video S1.** Swimming of a morphostatic specimen and a very late divider of *Schmidingerella* prior and after cell division under the stereo microscope, using dark field illumination.

**Video S2.** Ventrolateral view of a *Schmidingerella* very late divider under the light microscope, using interference contrast optics.

**Video S3.** Detail of a *Schmidingerella* very late divider under the light microscope, using interference contrast optics at 2000× magnification.

**Video S4.** Cell division and lorica‐forming material release by three proters of *Schmidingerella* under the light microscope, using interference contrast optics.

**Video S5.** Release of lorica‐forming material by three *Schmidingerella* proters under the light microscope, using interference contrast optics.

**Video S6.** Swimming and first phase of lorica formation by a *Schmidingerella* proter under the stereo microscope, using dark field illumination and an external LED light source.

## Data Availability

The data that support the findings of this study are available from the corresponding author upon reasonable request.
