# Supplementary material for: How Single Cells Form Shells: Maturation and Secretion of Lorica‐Forming Material in the Tintinnid Schmidingerella (Alveolata, Ciliophora)
Source: J Eukaryot Microbiol. 2025 Jul 8;72(4):e70025. doi: 10.1111/jeu.70025 (PMC12235346; doi:10.1111/jeu.70025)
Supplement: Supplementary file 1 — Table S1. Morphometrics of lorica‐forming material granules in cryofixed and chemically fixed Schmidingerella dividers. Table S2. TukeyHSD post hoc test results on lorica‐forming material granules linked with Figure S3. Figure S1. Ultrathin sections of cryofixed and chemically fixed Schmidingerella late dividers showing the co‐occurrence of different lorica‐forming material granule stages. Figure S2. Scatterplot and boxplots showing the relationship between the maximum diameter and the number of subunits in morula‐shaped lorica‐forming material granules of cryofixed and chemically fixed Schmidingerella late dividers. Figure S3. Box plots of the maximum diameters for three lorica‐forming material granule types (morula‐shaped, small mature, large mature) in cryofixed and chemically fixed Schmidingerella late dividers. Figure S4. Ultrathin sections of compact morula‐shaped lorica‐forming material granules in cryofixed and chemically fixed Schmidingerella late dividers. Figure S5. Histograms and cluster analyses of mature lorica‐forming material granule dimensions in cryofixed and chemically fixed Schmidingerella late dividers. Figure S6. Series of three longitudinal ultrathin sections through the cluster of mature lorica‐forming material granules in a cryofixed Schmidingerella late divider from the cluster’s center to its margin. Figure S7. Series of three longitudinal ultrathin sections through the cluster of mature lorica‐forming material granules in a chemically fixed Schmidingerella late divider from the cluster’s centre to its margin. Figure S8. Ultrathin longitudinal sections showing the arrangement of the mature lorica‐forming material granules in cryofixed and chemically fixed Schmidingerella late dividers. Figure S9. Analyses of two series each comprising three longitudinal ultrathin sections through the cluster of mature lorica‐forming material granules in a cryofixed and a chemically fixed Schmidingerella late divider, respectively. Figure S10. Electron ener [file JEU-72-e70025-s007.pdf]

Supporting Information

How Single Cells form Shells: Maturation and Secretion of Lorica-forming Material in the Tintinnid *Schmidingerella* (Alveolata, Ciliophora)

Maximilian H. Ganser[a], Birgit Weißenbacher[a], Sabine Agatha[a]

[a] Department of Environment & Biodiversity, University of Salzburg, 5020 Salzburg, Austria

Tables

TABLE S1 Morphometrics of lorica-forming material granules in cryofixed and chemically fixed *Schmidingerella* dividers.

TABLE S2 TukeyHSD post-hoc test results on lorica-forming material granules linked with Figure S3.

Figures

FIGURE S1 Ultrathin sections of cryofixed and chemically fixed *Schmidingerella* late dividers showing the co-occurrence of different lorica-forming material granule stages.

FIGURE S2 Scatterplot and boxplots showing the relationship between the maximum diameter and the number of subunits in morula-shaped lorica-forming material granules of cryofixed and chemically fixed *Schmidingerella* late dividers.

FIGURE S3 Box plots of the maximum diameters for three lorica-forming material granule types (morula-shaped, small mature, large mature) in cryofixed and chemically fixed *Schmidingerella* late dividers.

FIGURE S4 Ultrathin sections of compact morula-shaped lorica-forming material granules in cryofixed and chemically fixed *Schmidingerella* late dividers.

FIGURE S5 Histograms and cluster analyses of mature lorica-forming material granule dimensions in cryofixed and chemically fixed *Schmidingerella* late dividers.

FIGURE S6 Series of three longitudinal ultrathin sections through the cluster of mature lorica-forming material granules in a cryofixed *Schmidingerella* late divider from the cluster's centre to its margin.

FIGURE S7 Series of three longitudinal ultrathin sections through the cluster of mature lorica-forming material granules in a chemically fixed *Schmidingerella* late divider from the cluster's centre to its margin.

FIGURE S8 Ultrathin longitudinal sections showing the arrangement of the mature lorica-forming material granules in cryofixed and chemically fixed *Schmidingerella* late dividers.

FIGURE S9 Analyses of two series each comprising three longitudinal ultrathin sections through the cluster of mature lorica-forming material granules in a cryofixed and a chemically fixed *Schmidingerella* late divider, respectively.

FIGURE S10 Electron energy loss spectroscopy (EELS) of lorica-forming material granules and the lorica wall in a cryofixed *Schmidingerella* late divider.

FIGURE S11 Scanning electron micrographs of *Schmidingerella* specimens from a different strain previously cultured in our lab and a *Favella* specimen.

FIGURE S12 Ultrathin sections of finished cryofixed loricae of *Schmidingerella*.

Videos

VIDEO S1 Swimming of a morphostatic specimen and a very late divider of *Schmidingerella* prior and after cell division under the stereo microscope, using dark field illumination. The cluster of lorica-forming material is highly refractile. Very late dividers swim in narrow spirals, frequently with back-and-forth movements, seemingly aiding in the separation of proter and opisthe. Just before cell division, the proter extends far beyond the lorica rim, orientating itself at an oblique to perpendicular angle relative to the opisthe's main cell axis.

VIDEO S2 Ventrolateral view of a *Schmidingerella* very late divider under the light microscope, using interference contrast optics. The opisthe's circular oral apparatus is ciliated but rather inactive. Pin-shaped cytoplasmic extensions (tentaculoids) and cytoplasmic strands (striae) longitudinally extending on the membranelles contain capsules and mucocysts (tintinnid extrusomes). The cluster of lorica-forming material is just underneath the proter's extremely long lateral ciliary field. Small and large granules of lorica-forming material can be distinguished. The outer lorica surface exhibits a reticulate pattern of ridges, while the inner surface is smooth.

VIDEO S3 Detail of a *Schmidingerella* very late divider under the light microscope, using interference contrast optics at 2,000× magnification. The cluster of lorica-forming material during the size-based sorting is shown, which results in the formation of a longitudinal strip of small granules embedded in large granules.

VIDEO S4 Cell division and material release by three proters of *Schmidingerella* under the light microscope, using interference contrast optics. As the dorsal cytoplasmic strand narrows over the course of a few minutes, the opisthe's oral primordium reorients nearly perpendicular to the main cell axis. After the transverse split, the remains of the cytoplasmic strand previously linking proter and opisthe are usually resorbed within a minute. Within the first two minutes after separation, a considerable amount of lorica-forming material is secreted, i.e., the small granules are secreted followed by the large ones. The just

extruded granules show for a very short period minute subunits resembling the morula-shaped granules before they start swelling, merging, and alveolarization. The movement of the cilia of the extremely long lateral field facilitate the contact between the granules and their subsequent mutual cohesion. Please, note that the specimens are unable to freely move and to generate a lorica.

VIDEO S5 Release of lorica-forming material by three *Schmidingerella* proters under the light microscope, using interference contrast optics. First, the small granules are secreted followed by the large ones. Minute indentations remain where the granules have been secreted. The just extruded granules show for a very short period minute subunits resembling the morula-shaped granules before they start swelling, merging, and alveolarization (Figure 9). The movement of the cilia of the extremely long lateral field facilitate the contact between the granules and their subsequent mutual cohesion. Please, note that the specimens are unable to freely move and to generate a lorica.

VIDEO S6 Swimming and first phase of lorica formation by a *Schmidingerella* proter under the stereo microscope, using dark field illumination and an external LED light source. The cluster of lorica-forming material is recognisable as a highly refractile oblique strip. First, the cell obtains an ellipsoidal shape. The material release is not recognisable at this magnification but the emergence of a refractile layer covering the cell surface (thimble-shaped roughcast) and the development of a posterior lorica process are visible. Only when the peduncle of the now somewhat asymmetrically obconical cell extends, the lorica wall becomes discernible and more prominent possibly due to a further swelling and alveolarization of the material. Unfortunately, the completion of the lorica by the addition of the anterior third of the final length could not be followed.

TABLES

TABLE S1 Morphometrics of lorica-forming material granules in cryofixed and chemically fixed *Schmidingerella* dividers.

Fixation	Type	Characteristics[a]	$\bar{x}$	M	SD	SE	CV	Min	Max	n
Cryofixed	Morula	Maximum diameter	883	923	294.5	34.7	33.4	157	1,749	72
	Mature, small		809	814	206.5	11.0	25.5	394	1,231	353
	Mature, large		1,565	1,535	258.9	15.0	16.5	1,120	2,538	299
Chemically fixed	Morula	Maximum diameter	1,382	1,482	517.7	143.6	37.5	460	2,052	13
	Mature, small		957	945	314.6	14.7	32.9	254	1,567	457
	Mature, large		2,132	2,083	405.5	22.6	19.0	1,504	3,271	323
Cryofixed	Morula	Number of subunits	4.5	4.5	2.4	0.3	54.1	1	10	72
Chemically fixed	Morula		12.3	14.0	5.1	1.4	41.1	3	20	13

[a] Measurements in nm. CV, coefficient of variation in %; M, median; Max, maximum; Min, minimum; n, number of granules investigated; SD, standard deviation; SE, standard error of arithmetic mean; $\bar{x}$, arithmetic mean.

TABLE S2 TukeyHSD post-hoc test results on lorica-forming material granules linked with Figure S3. diff, mean difference (rounded) between the two compared groups; lwr and upr, lower and upper bounds of the 95% confidence interval for the mean difference; p adj, adjusted p-value for the comparison.

Size comparisons	diff	lwr	upr	p adj
Mature, cryofixed: small vs. large granules	-756	-825	-687	< 0.05
Mature, chemically fixed: small vs. large granules	-1,175	-1,239	-1,112	< 0.05
Mature, small: cryofixed vs. chemically fixed granules	-148	-210	-86	< 0.05
Mature, large: cryofixed vs. chemically fixed granules	-567	-638	-497	< 0.05
Morula-shaped: cryofixed vs. chemically fixed granules	-499	-763	-234	< 0.05
Cryofixed: small mature vs. morula-shaped granules	-74	-187	39	0.43
Cryofixed: large mature vs. morula-shaped granules	682	567	797	< 0.05
Chemically fixed: small mature vs. morula-shaped granules	-425	-672	-179	< 0.05
Chemically fixed: small mature vs. morula-shaped granules	-425	-672	-179	< 0.05

FIGURES

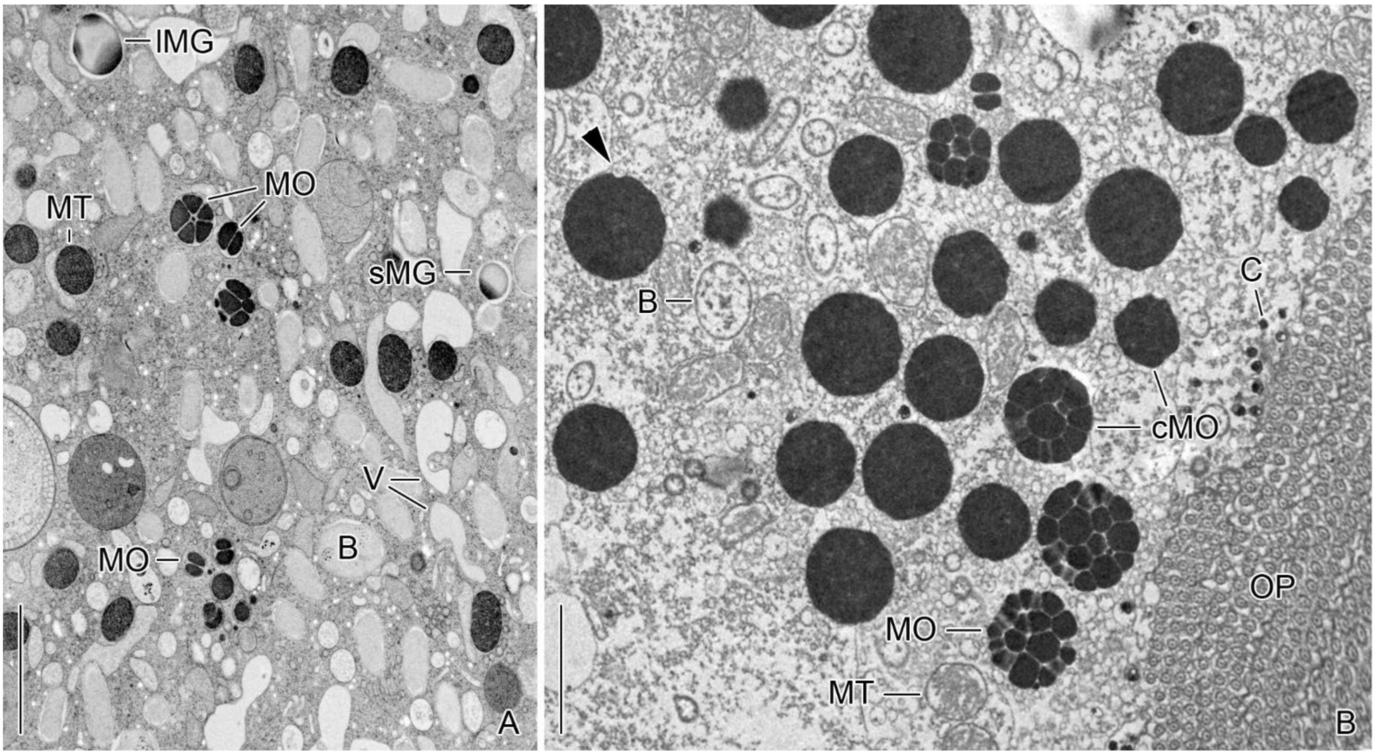

FIGURE S1 Ultrathin sections of cryofixed (A) and chemically fixed (B) *Schmidingerella* late dividers showing the co-occurrence of different lorica-forming material granule stages. Arrowhead (B) denotes the protrusion of a mature granule. B, bacteria; C, capsules (tintinnid extrusomes; see Ganser et al., 2023); cMO, compact morula-shaped granules; lMG, large mature granule; MO, morula-shaped granules; MT, mitochondria; OP, oral primordium (developing oral ciliature of opisthe); sMG, small mature granule; V, vesicles. Scale bars 2.5 µm.

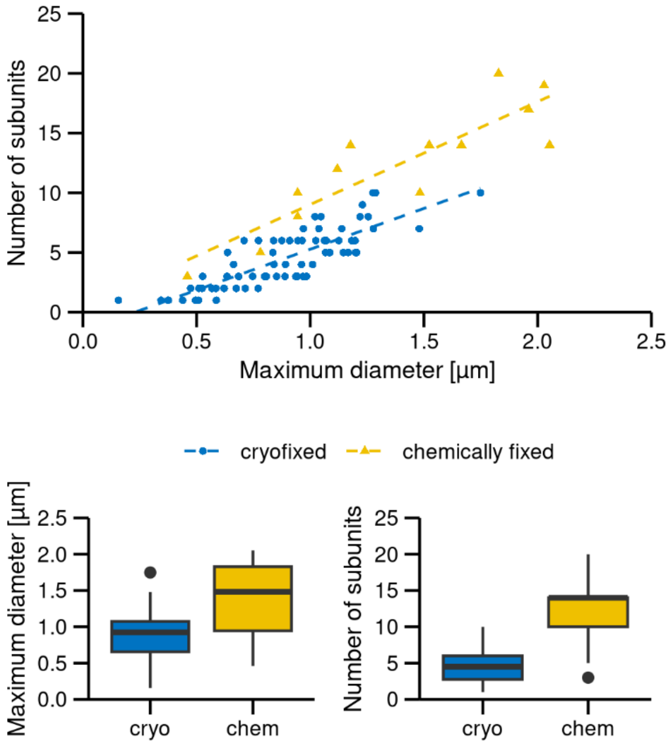

FIGURE S2 Scatterplot and boxplots showing the relationship between the maximum diameter and the number of subunits in morula-shaped lorica-forming material granules of cryofixed (blue; n = 72) and chemically fixed (orange; n = 13) *Schmidingerella* late dividers. The maximum diameter of the morula-shaped granules increases with the number of constituting subunits. In comparison, morula-shaped granules are larger and consist of more subunits in chemically fixed than in cryofixed specimens.

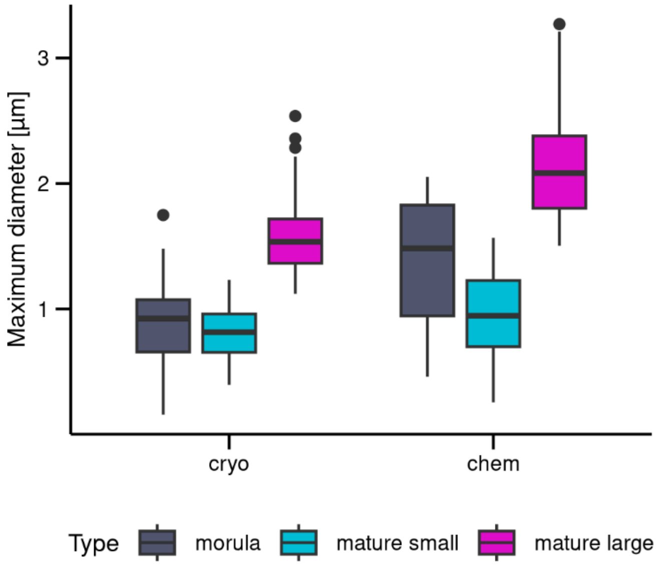

FIGURE S3 Box plots of the maximum diameters for three lorica-forming material granule types (morula-shaped, small mature, large mature) in cryofixed (cryo) and chemically fixed (chem) *Schmidingerella* late dividers. The mature granules of the respective size class are significantly larger in the chemically fixed material than in the cryofixed one (cp. Table S2). The mean size difference of the morula-shaped and small mature granules in cryofixed material is insignificant, whereas they are on average much smaller than the large mature granules. Horizontal boxplot line, median.

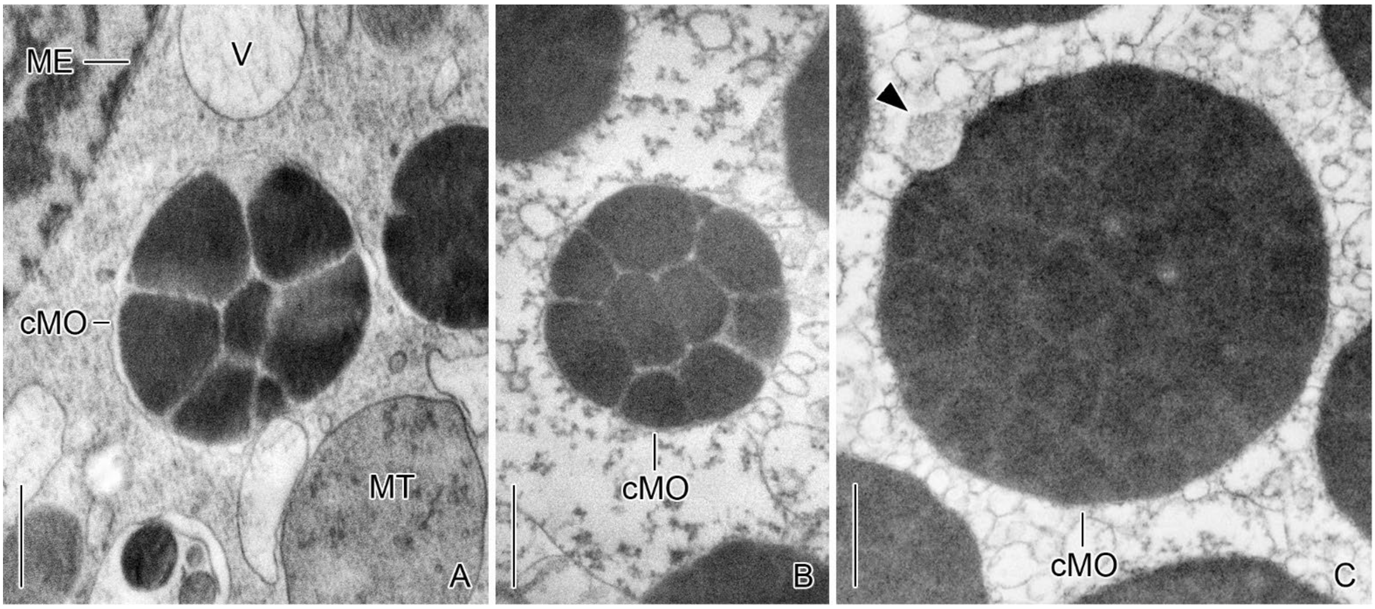

FIGURE S4 Ultrathin sections of compact morula-shaped lorica-forming material granules in cryofixed (A) and chemically fixed (B, C) *Schmidingerella* late dividers. (A, B) Typical granules with narrow gaps between subunits. (C) Granule combining features of the compact morula-shaped granules (thin electron-transparent lines) with characters of mature granules, namely, a protrusion (arrowhead) and electron-transparent spots. cMO, compact morula-shaped granules; ME, macronuclear envelope; MT, mitochondrium; V, vesicle. Scale bars 500 nm.

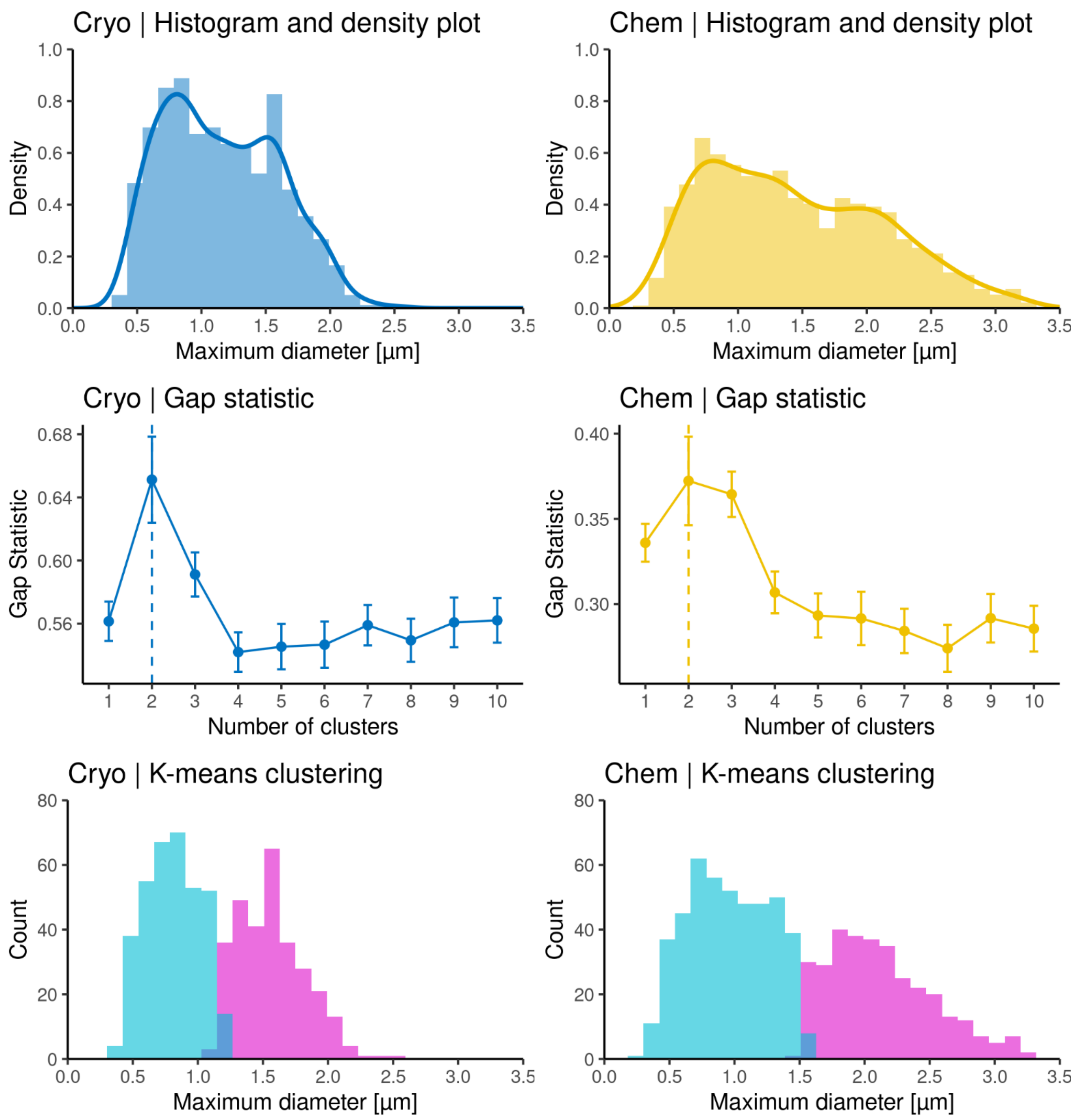

FIGURE S5 Histograms and cluster analyses of mature lorica-forming material granule dimensions in cryo-fixed (left column) and chemically fixed (right column) *Schmidingerella* late dividers. The gap statistic method suggested an optimal separation of the mature granules into two size classes (cyan = small; magenta = large), which hardly overlap in their maximum diameters (K-means clustering).

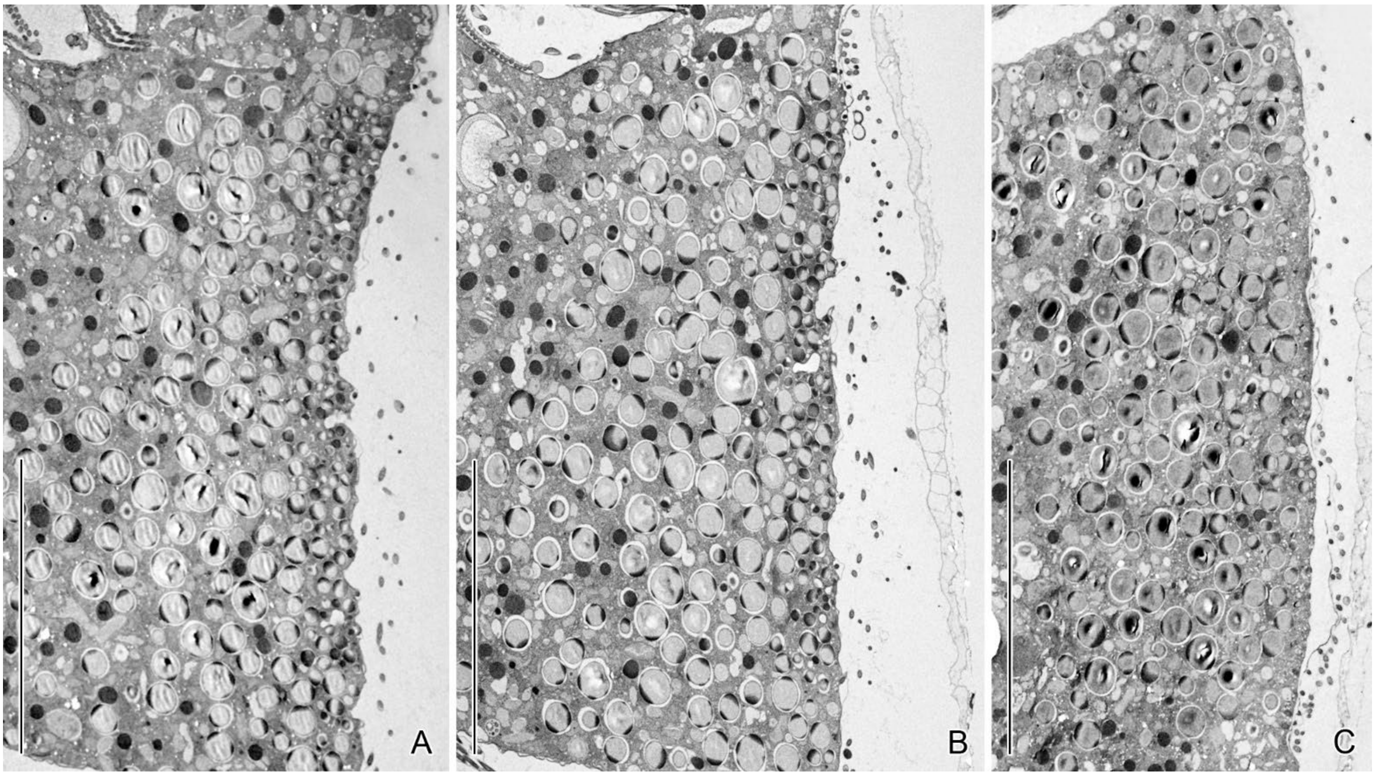

FIGURE S6 Series of three longitudinal ultrathin sections through the cluster of mature lorica-forming material granules in a cryofixed *Schmidingerella* late divider from the cluster's centre (A) to its margin (C). These sections were analysed for inferring the distribution patterns of the small and large mature granules in the cluster (Figures 5 and S9). Scale bars 15 µm.

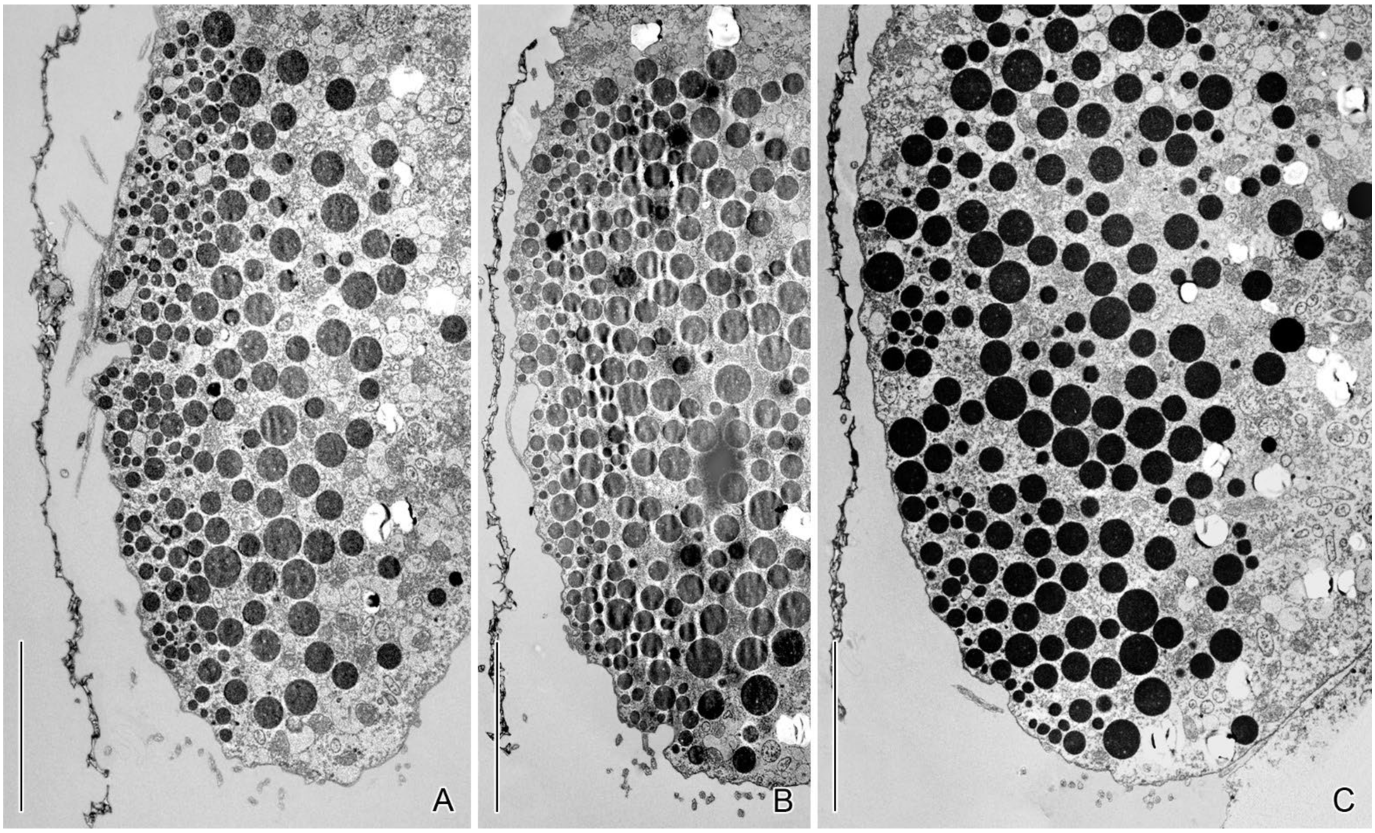

FIGURE S7 Series of three longitudinal ultrathin sections through the cluster of mature lorica-forming material granules in a chemically fixed *Schmidingerella* late divider from the cluster's centre (A) to its margin (C). These sections were analysed for inferring the distribution patterns of the small and large mature granules in the cluster (Figures 5 and S9). Scale bars 10 µm.

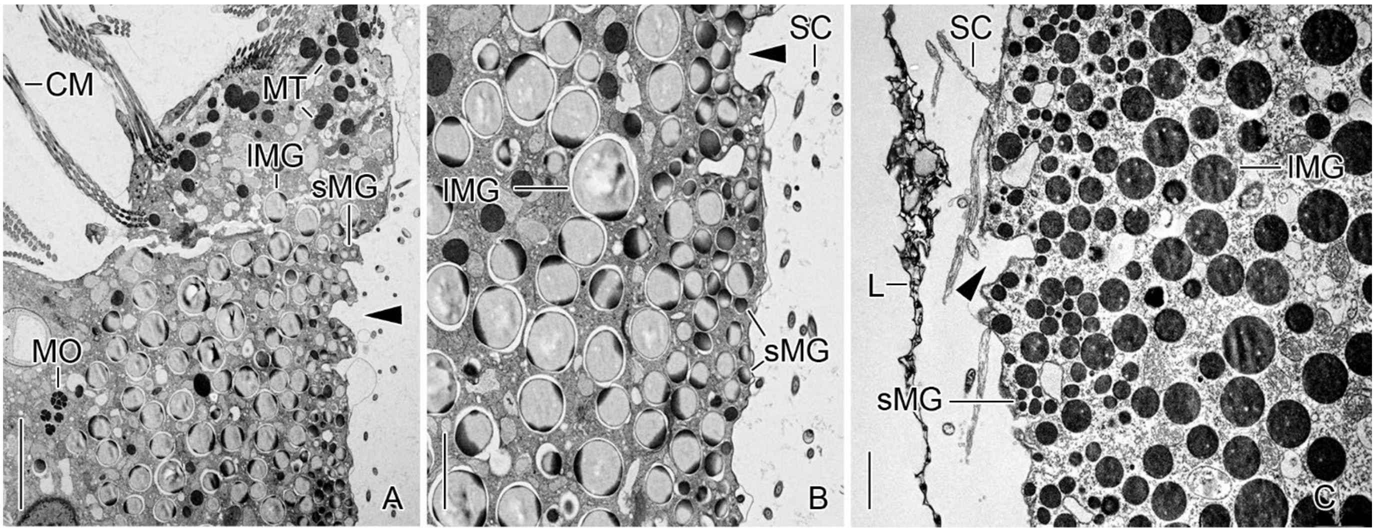

FIGURE S8 Ultrathin longitudinal sections showing the arrangement of the mature lorica-forming material granules in cryofixed (A, B) and chemically fixed (C) *Schmidingerella* late dividers. The small granules form a peripheral longitudinal strip just underneath the lateral ciliary field and are embedded in large granules. The cell surface displays several invaginations (arrowheads). CM, collar membranelles; L, lorica; lMG, large mature granules; MO, morula-shaped granules; MT, mitochondria; SC, somatic cilia; sMG, small mature granules. Scale bars 5 µm (A), 2.5 µm (B, C).

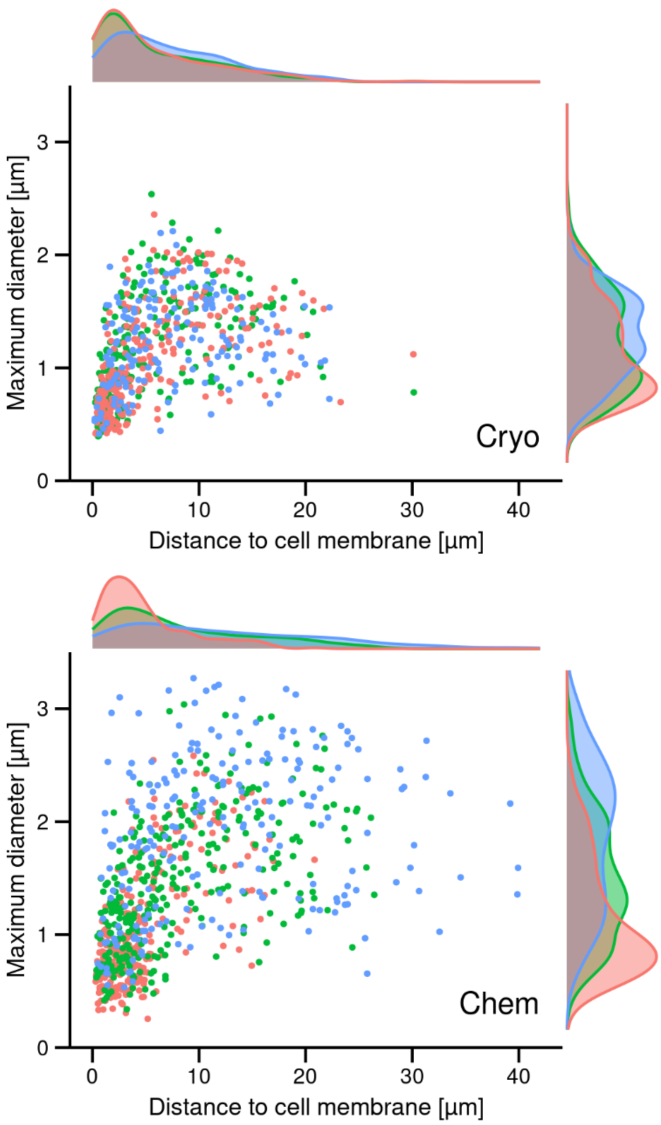

FIGURE S9 Analyses of two series each comprising three longitudinal ultrathin sections through the cluster of mature lorica-forming material granules in a cryofixed (top) and a chemically fixed (bottom) *Schmidingerella* late divider, respectively (Figures S6A-C and S7A-C). The small mature granules are close underneath the cell cortex and rather restricted to the cluster's central portion, where they form a longitudinal strip. Large mature granules mainly occur laterally and proximally of this strip. The dots and density curves are colour-coded: section 1 (centre), red; section 2, green; section 3 (margin), blue.

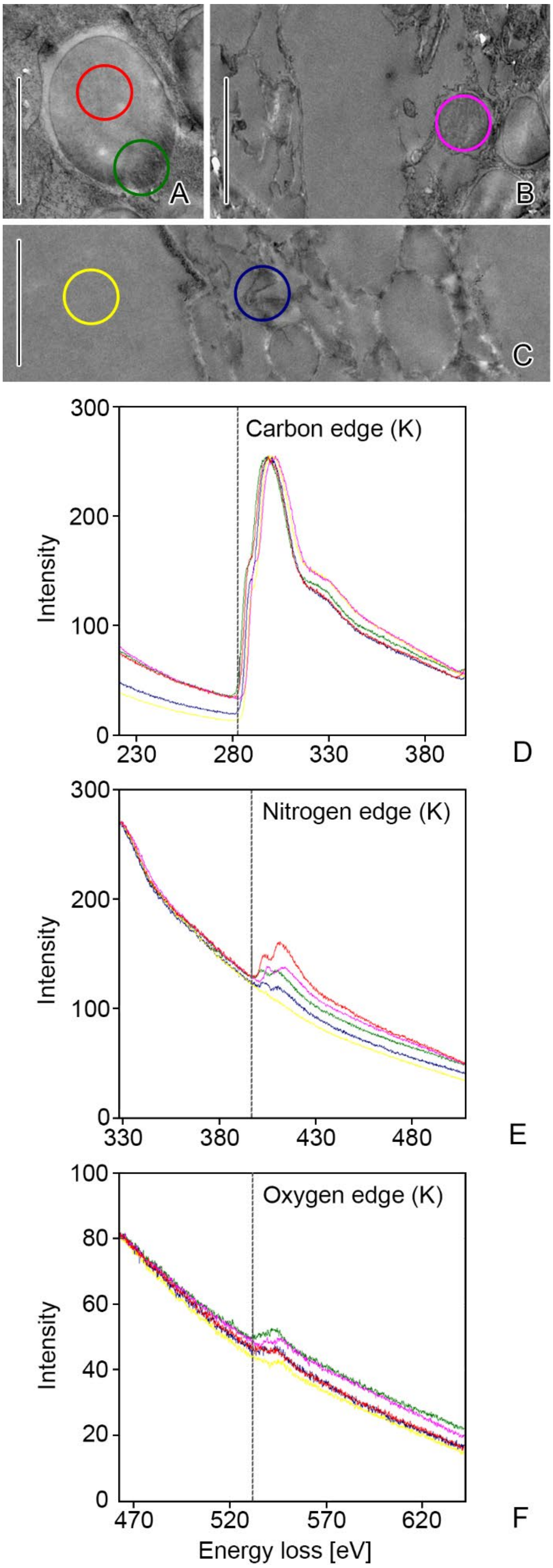

FIGURE S10 Electron energy loss spectroscopy (EELS) of lorica-forming material granules and the lorica wall in a cryofixed *Schmidingerella* late divider. (A-C) Ultrathin sections showing the measured spots. (A) Large mature granule with its centre (red) and cap region (green). (B) Small mature granule (magenta). (C) Lorica wall (blue) and embedding medium (yellow; reference). (D-F) Measurements near the specific energy loss edges of carbon (D), nitrogen (E), and oxygen (F). Scale bars 1 µm.

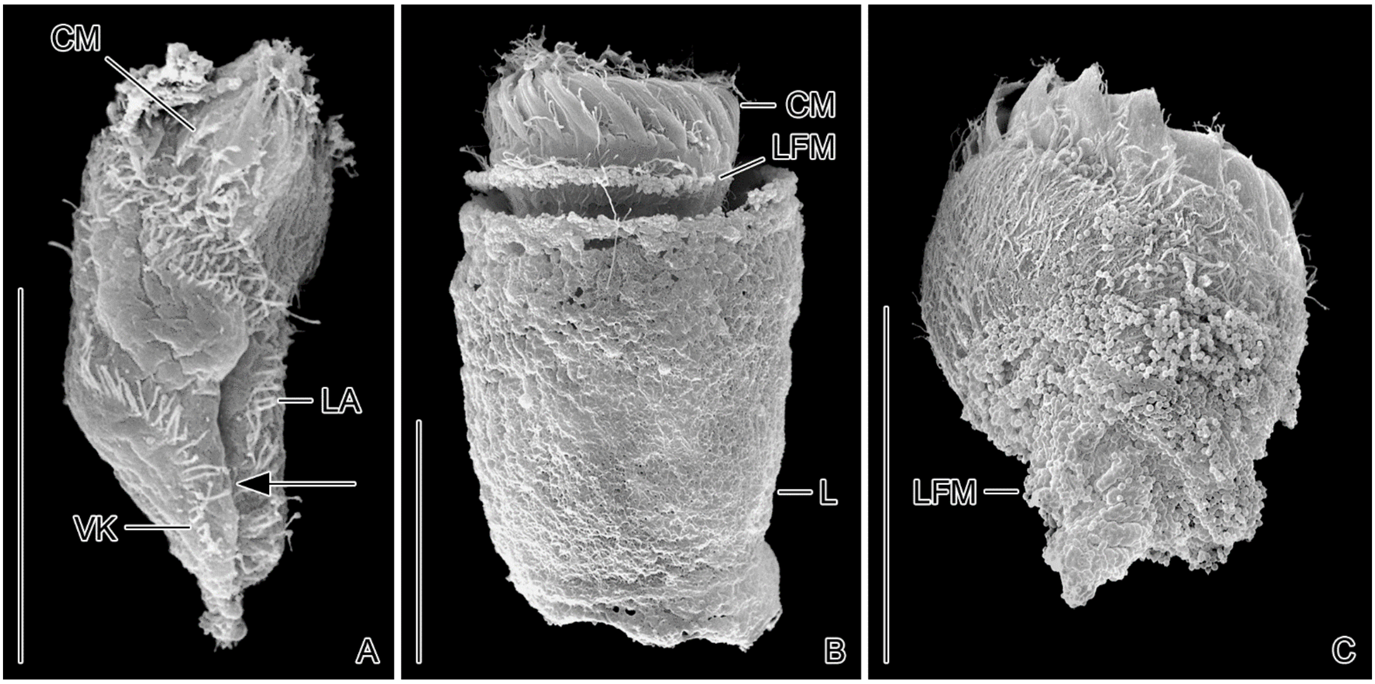

FIGURE S11 Scanning electron micrographs (B, C from Agatha et al., 2013) of *Schmidingerella* specimens from a different strain previously cultured in our lab (A, B) and a *Favella* specimen (C). (A) Ventral view of a postdivider showing the longitudinal furrow (arrow) which supposedly results from a massive release of lorica-forming material. (B) Lateral view showing a strand of just secreted and thus not fully merged LFM granules somewhat apart from the lorica rim (artificially detached or not yet attached). (C) Oblique posterior view. In the anterior cell portion, a shallow furrow might be the secretion site of the LFM which already covers the posterior cell portion as a layer of partly fused granules. CM, collar membranelles; L, lorica; LA, lateral ciliary field; LFM, lorica-forming material; VK, ventral kinety. Scale bars 40 µm.

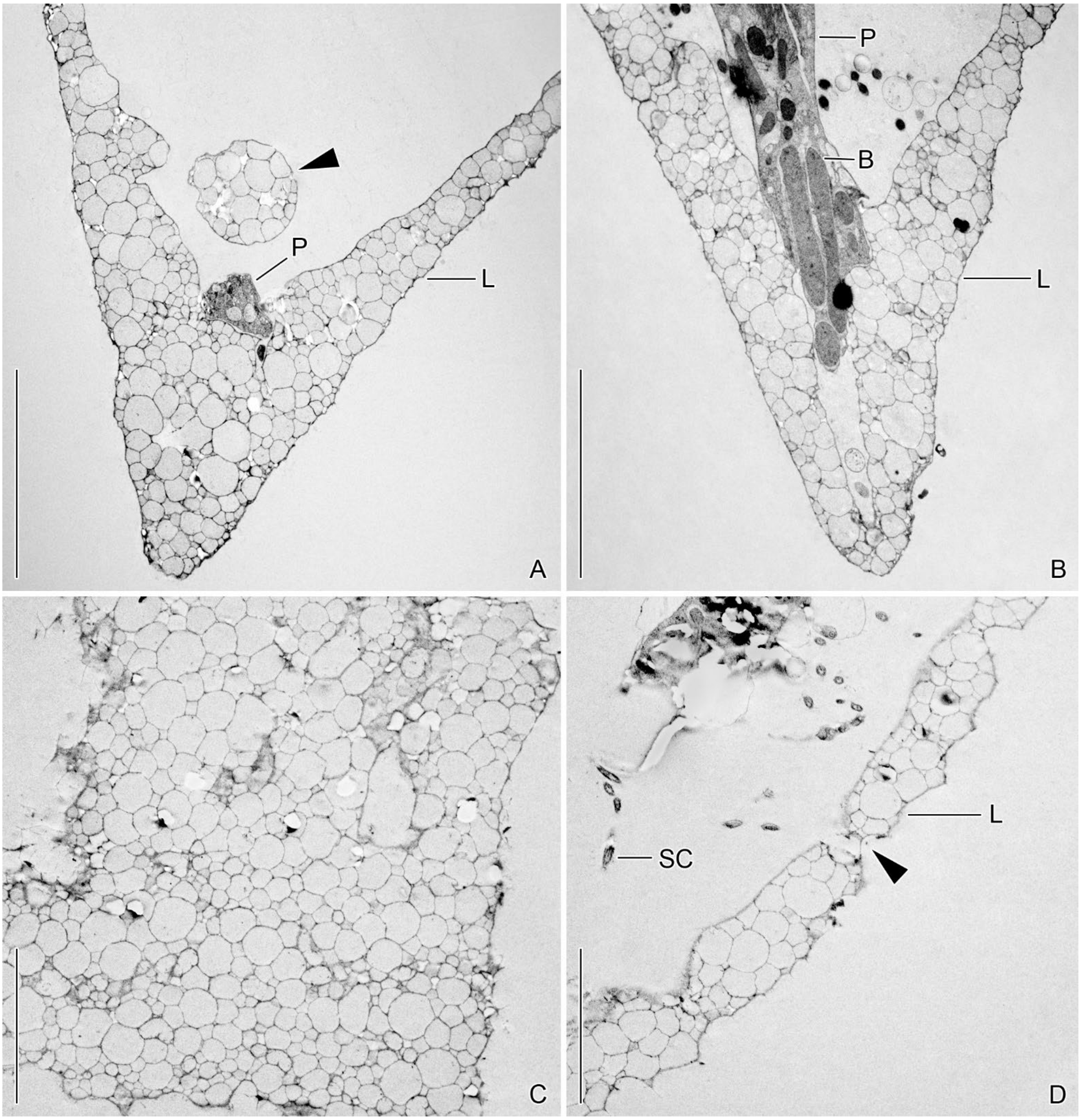

FIGURE S12 Ultrathin sections of finished cryofixed loricae of *Schmidingerella*. (A, B) Longitudinal sections of the same posterior process. An inner protrusion is occasionally observed close to the process base (A; arrowhead). The cell's contractile peduncle extends into the canal of the lorica's posterior process (B). In this specimen, it contains several bacteria. (C) Tangential section of lorica wall showing the irregular alveoli. (D) Longitudinal section of lorica wall showing the alveoli, an opening (arrowhead), and surface ridges. B, bacteria; L, lorica; P, peduncle; SC, somatic cilia. Scale bars 10 µm (A, B), 5 µm (C, D).

REFERENCES

Agatha, S., Laval-Peuto, M. & Simon, P. (2013) The tintinnid lorica. In: Dolan, J.R., Montagnes, D.J.S., Agatha, S., Coats, D.W. & Stoecker, D.K. (Eds.) *The Biology and Ecology of Tintinnid Ciliates: Models for Marine Plankton.* Oxford, Chichester: John Wiley & Sons, Ltd., pp. 17–41.

Ganser, M.H., Bartel, H., Fedrizzi, M. & Agatha, S. (2023) A comparative ultrastructural study on the nanoscale extrusomes of tintinnids (Alveolata, Ciliophora, Spirotricha) and their phylogenetic significance. *European Journal of Protistology,* 87, 125953.
